# A Faithful Discretization of Verbose Directional Transforms

**DOI:** 10.1007/s00454-025-00791-w

**Published:** 2025-11-25

**Authors:** Brittany Terese Fasy, Samuel Micka, David L. Millman, Anna Schenfisch, Lucia Williams

**Affiliations:** 1 School of Computing, Montana State U, Bozeman, Montana USA; 2 Department of Mathematical Sciences, Montana State U, Bozeman, Montana USA; 3 Mathematics & Computer Science Department, Western Colorado U, Gunnison, Colorado USA; 4 Blocky Inc, Bozeman, Montana USA; 5 KTH Royal Intitute of Tech, Stockholm, Sweden; 6 University of Montana, Missoula, MT USA

**Keywords:** Immersed simplicial complexes, Persistence diagrams, Betti functions, Euler characteristic curves, Directional transforms, Reconstruction, Shape representation, 52C45, 51M20, 57Q99, 46M20

## Abstract

The persistent homology transform, Betti function transform, and Euler characteristic transform represent a shape with a multiset of persistence diagrams, Betti functions, or Euler characteristic functions, respectively, parameterized by the sphere of directions in the ambient space. In this work, we give the first explicit construction of finite sets of directions discretizing the verbose variants of these transforms and show that such discretizations faithfully represent the underlying shape. Our discretization, while exponential in the dimension of the shape, does not depend on any restrictions on the particular immersion beyond general position, and is stable with respect to various perturbations.

## Introduction

Collections of topological descriptors are both empirically and theoretically useful for analyzing data and differentiating shapes [4, 18, 23, 25, 32, 35, 39, 41]. In [37], Turner et al. define the persistent homology transform (PHT) and Euler characteristic (function) transform (ECT), which map a geometric simplicial complex in $\mathbb {R}^{d}$ to a family of persistence diagrams and Euler characteristic functions parameterized by the directions in $\mathbb {S}^{d-1}$. They show that, for $d=2,3$, these *uncountably infinite* parameterized families are *faithful* representations of shape, meaning that no two distinct shapes have the same family of directional persistence diagrams or Euler characteristic functions. A few years later, this result was proven for general *d* in [17], by showing that injectivity of the ECT is a special case of the inversion theorem of Schapira [33], which is a perspective more aligned with the closely related field of tomography; see also [21, 24]. Recent related work explores both properties and variations of directional transforms [7, 38]. In particular, several research groups independently observed. In addition, several research groups independently observed that there exist *finite* faithful representations for various types of simplicial and cubical complexes [2, 3, 5, 11, 30, 31].

Meanwhile, researchers are already applying the PHT and the closely related verbose PHT (VPHT) to represent various types of data sets in machine learning and statistical pipelines [5, 10, 20, 22, 27, 37]. In many of these papers, directions are sampled uniformly, which, without additional assumptions on the underlying shape, has no guarantee of resulting in a faithful set. On the other hand, some theoreteical results only apply to a sub-class of simplicial complexes and the faithful sets are large. A main result of [11], Theorem 7.14, bounds the number of concise (i.e., not verbose) Euler characteristic functions or persistence diagrams needed to form a faithful discretization, so long as the underlying simplicial complex *K* satisfies additional constraints, relating to its its “flatness” and complexity. Because of the dependence on the properties of the embedding of *K*, it is easy to identify families of simplicial complexes that make the bound of [11] arbitrarily large. For instance, consider a path of length two embedded in Euclidean space. By moving the vertices closer and closer to colinear, the bound on the number of concise persistence diagrams needed to represent the path using the methods of [11] grows arbitrarily large; see Example 42 in Appendix A. Sensitivity to the flatness of *K* is intimately related to using the classic *concise* versions of descriptors rather than verbose versions of descriptors. Roughly speaking, unlike concise descriptors, verbose descriptors do not omit the record of instantaneous changes to topological invariants during a filtration, which has a significant impact on the behavior of faithful sets. As we show in Example 42, for the aforementioned family of increasingly flat path graphs, the size of a faithful discretization made up of verbose descriptors does *not* change. Furthermore, while results in in [11] broadly describe conditions needed to form a finite faithful set, there are no explicit algorithms for finding such a set, and thus, the results remain disconnected from practical use.

In this paper, we provide an explicit discretization of verbose directional transforms whose size remains constant as the shape is perturbed, extending work that began in [3, 14]. Belton et al. began in the setting of embedded plane graphs with $n_0$ vertices, and gave a faithful discretization of the VPHT using only $\Theta (n_0^2)$ verbose persistence diagrams [3, Theorems 15 and 16]. In [14, Theorem 10], we remove the planar condition and improve this bound to $O(d + n_1 \log n_0)$, where $n_1$ is the number of edges in the graph. To show that these discretizations are indeed faithful, both [3] and [14] use the proof method of *reconstructing* the shape from the set of diagrams. This work takes the natural next step of using reconstruction to give the first explicit faithful discretization of the VPHT, the VBT, and the VECT, for general simplicial complexes immersed in $\mathbb {R}^d$. Roughly speaking, our discretization is made up of descriptors corresponding to directions perpendicular to and “tilted around” each maximal simplex of an unknown simplicial complex *K*, and has size $O(n2^{{\dim (K)}}+d)$, where *n* is the number of simplices in the unknown simplicial complex. Our first main result is Theorem 14, which uses reconstruction to show that a set of descriptors satisfying certain conditions is faithful. We then go on to give explicit algorithms for constructing such a set, which culminates in our second main result, Theorem 35. Importantly, the size of our discretization does not depend on any restrictions on the underlying simplicial complex beyond standard general position assumptions.

## Background Definitions

We assume that the reader is familiar with homology groups (denoted $H_*$) and their Betti numbers (denoted $\beta _*$). For a more complete discussion on foundational computational topology, we refer the reader to [8, 12].

### Directional Transforms

In this section, we provide definitions fundamental to directional transforms.

***Simplices and Simplicial Complexes***

Let $k,d \in \mathbb {N}$. A *(geometric)* *k*-*simplex* $\sigma$ is the convex hull of a set of $k+1$ affinely independent points in $\mathbb {R}^d$, denoted $\sigma =[v_0,v_1, \ldots , v_k]$. Each of these points is called a *vertex*, and we denote the vertex set of $\sigma$ by $\textrm{verts}(\sigma )$; at times, we use $\sigma$ in place of $\textrm{verts}(\sigma )$ when using $\textrm{verts}(\sigma )$ would make equations cumbersome to read. We call *k* the *dimension* of $\sigma$, and write $\dim (\sigma ):=k$. For another simplex $\tau$, we say that $\tau$ is a *face* of $\sigma$ and $\sigma$ is a *coface* of $\tau$ if $\emptyset \ne \textrm{verts}(\tau ) \subseteq \textrm{verts}(\sigma )$; we denote this relation by $\tau \preceq \sigma$. If $\tau \preceq \sigma$ but $\tau \ne \sigma$, then $\tau$ is called a proper face of $\sigma$, denoted $\tau \prec \sigma$. If $\sigma$ is not the proper face of any simplex, we say that $\sigma$ is *maximal*.

A *GP-immersed simplicial complex* $K$ is a finite set of geometric simplices such that the vertex set of $K$ is in general position.^1^ We topologize *K* with the Alexandroff topology. We denote the set of *k*-simplices in $K$ by $K_k$ and the number of simplices in $K_k$ by $n_k$. We let $n=\sum _{k\in \mathbb {N}} n_k$.

***Filtrations and Persistent Homology***

Let $f :K\rightarrow \mathbb {R}$ be such that, for each pair of simplices $\tau \prec \sigma \in K$, we have $f( \tau ) \le f(\sigma )$. Each sublevel set $F_x:=f^{-1}(-\infty ,x]$ with $x\in \mathbb {R}$ is a simplicial complex and we call *f* a *filter* function. The parameterized sequence of subcomplexes $\{ F_x \}_{x \in \mathbb {R}}$, along with inclusions $F_x \subseteq F_{y}$ for all $x \le y$, is the *filtration of* *K* *with respect to* *f*. This filtration realizes at most $n+1$ distinct complexes: the empty set and $F_{f(\sigma )}$ for each $\sigma \in K$.

One filtration of interest, an *index filtration*, arises when *f* is an *index filter*, that is *f* is injective and $\text {im}(f) = \{1,2,\ldots , n \}$. Notably, in an index filtration, simplices of *K* are added one by one. If *K* is GP-immersed in $\mathbb {R}^d$, another filtration of interest is the *lower-star filtration* with respect to a direction $s\in \mathbb {S}^{d-1}$. For each vertex $v \in K_0$, the height of *v* in direction $s$ is given by the dot product, $s\cdot v$. The lower-star filter function in direction $s$, denoted $h_s: K\rightarrow \mathbb {R}$, defines the “height” of each simplex in $K$ where $h_s(\sigma ) = \max \{s\cdot v ~\vert ~v \in \textrm{verts}(\sigma )\}$, i.e., $h_{s}(\sigma )$ is the height of the highest vertex in $\sigma$ with respect to $s$. The lower-star filtration is the filtration of *K* with respect to $h_{s}$. For $x,y \in \mathbb {R}\cup \{\pm \infty \}$ such that $x \le y$, we have $h_s^{-1}(-\infty ,x]=h_s^{-1}(-\infty ,y]$ if and only if no vertex has height in the interval (*x*, *y*].

Applying the homology functor to a filtration $\{F_x \}_{x \in \mathbb {R}}$, we obtain the *persistence module* $\{H(F_x) \}_{x \in \mathbb {R}}$. Here, we assume that homology is computed using field coefficients (e.g., $\mathbb {Z}_2$). Then, each $H(F_x)$ is a graded vector space $\oplus _{k \in \mathbb {N}} H_k(F_x)$. For each $k \in \mathbb {N}$ and for each $x \le y$, the inclusion of simplicial complexes $F_x \subseteq F_{y}$ induces a linear map $f_{k,x,y} :H_k(F_x) \rightarrow H_k(F_{y})$. Let $\beta _{k,x,y}=\beta _{k,x,y}(f)$ denote the rank of $f_{k,x,y}$. We define the *k*-multiplicity at (*x*, *y*) as

1 $$\begin{aligned} \mu _k(x,y):= {\left\{ \begin{array}{ll} \lim _{\varepsilon \rightarrow 0} \left( \beta _{k,x,y-\varepsilon } - \beta _{k, x,y} - \beta _{k, x-\varepsilon ,y-\varepsilon } + \beta _{k, x-\varepsilon ,y}\right) & \text {if } x<y \\ 0 & \text {else}. \end{array}\right. } \end{aligned}$$

Then, for $k \in \mathbb {N}$, the *k*-*dimensional persistence diagram* is the following multiset:

2 $$\begin{aligned} \mathcal {D}_{f,k}:= \{ (x,y)^{\mu _k(x,y)} \}_{(x,y) \in \overline{\mathbb {R}}^2}, \end{aligned}$$

where $(a,b)^m$ denotes *m* copies of the point (*a*, *b*), and $\overline{\mathbb {R}} := \mathbb {R}\cup \{ \pm \infty \}$. In other words, each $(x,y)\in \mathcal {D}_{f,k}$ represents a *k*-dimensional homology generator $\alpha$ that is born at *x* (that is, $[\alpha ] \in H_k(F_x)$ but $[\alpha ] \notin \text {im}(f^{x-\varepsilon ,x}_k)$) and dies going into *y* (that is, *y* is the smallest index such that there exists $[\alpha '] \ne [\alpha ] \in H_k(F_{x})$ with $[\alpha ]=[\alpha ']$ in $H_k(F_y)$). The *persistence diagram* is the graded multiset: $\mathcal {D}_{f}:= \sqcup _{k \in \mathbb {N}} \mathcal {D}_{f,k}$.

***Verbose Persistence Diagram***

Because simplices can have the same height in a filtration, it is possible that the birth and the death of a *k*-cycle happen simultaneously, in which case, that cycle is not represented in the persistence diagram. However, having every simplex “appear” in the persistence diagram is helpful. Thus, we introduce *verbose persistence diagrams* (VPDs). Given a filter function $f :K \rightarrow \mathbb {R}$, an index filter $f' :K \rightarrow \mathbb {N}$ is called *compatible with* *f* if for all $\tau , \sigma \in K$ such that $f(\tau ) \le f(\sigma )$, we have $f'(\tau ) \le f'(\sigma )$.

#### Definition 1

*(Verbose Persistence Diagram)* Given a filter $f :K\rightarrow \mathbb {R}$, let $f'$ be a compatible index filtration. For $k \in \mathbb {N}$, the *k*-*dimensional verbose persistence diagram* is the following multiset:

3 $$\begin{aligned} \widetilde{\mathcal {D}}_{f, k} := \left\{ \big ( f(f'^{-1}(i)), f(f'^{-1}(j)) \big ) \right\} _{(i,j) \in \mathcal {D}_{f',k}}, \end{aligned}$$

where $f(\emptyset ):=\infty$ and $f'(\emptyset ):=\infty$ for convenience. The *verbose persistence diagram* (VPD) of *f* is the graded multiset of all *k*-dimensional verbose persistence diagrams: $\widetilde{\mathcal {D}}_{f}:= \sqcup _{k \in \mathbb {Z}} \widetilde{\mathcal {D}}_{f, k}$.

Because the filter function $f'$ in the definition above need not be unique, it is not immediately clear that this definition is well-defined. A proof that VPDs are, in fact, well-defined is found in Appendix B.

The VPD carries more information than the persistence diagram (as the height of each simplex is a coordinate of a persistence point). Yet they do not require extra work to compute; algorithms for computing PDs compute VPDs as an intermediate step (see, e.g., [12, Chapter VII.2]). The VPD has been used in several contexts already. For example, the definition of PD in McCleary and Patel [28] is the same as our definition of VPD. Usher and Zhang define verbose barcodes via the lens of filtered chain complexes in [40]. This perspective is also taken in, e.g., [6, 29], where instantaneous or length-zero bars are referred to as “ephemeral.” In other sources, “verbose” is sometimes replaced by the word “augmented,” e.g., [3, 14, 30]. We only use verbose persistence diagrams in this paper, so we use “diagram” as shorthand for “VPD.”

***Betti Functions***

The $k^{\text {th}}$ Betti number of a simplicial complex $K$ is the rank of the *k*-dimensional homology group of $K$, and is denoted  $\beta _k(K) = \text {rank}(H_k(K))$. Measuring this quantity as a filtration parameter changes and for all $k\in \mathbb {Z}$ gives rise to *Betti function*.

#### Definition 2

*(Betti Function (BF) and Verbose BF)* Given a filter $f :K\rightarrow \mathbb {R}$, the $k^{\text {th}}~Betti~function~(k^{\text {th }}BF)$ is the step function $\beta _{f,k}: \mathbb {R}\rightarrow \mathbb {Z}$, where

4 $$\begin{aligned} \beta _{f,k}(t) := \beta _k \left( f^{-1}(-\infty ,t]\right) . \end{aligned}$$

The collection of all Betti functions, indexed by dimension, $\beta _{f}:= \{ \beta _{f,k}\}_{k \in \mathbb {N}}$, is referred to as the *Betti function*.

Suppose $f'$ is an index filter compatible with *f*. Let $\sigma \in K$. If $\sigma$ appearing in the index filtration of $f'$ corresponds with $\beta _k$ increasing (respectively, decreasing), then we say $\sigma$ is *positive* (resp., *negative*) *for* $\beta _k$ with respect to $f'$. The set of positive (resp., negative) simplices is denoted $K_k^+ \subseteq K_k$ (and $K_{k+1}^- \subseteq K_{k+1}$). Then, we define the *kth verbose Betti function*, $\widetilde{\beta }_{f, k}: \mathbb {R}\rightarrow \mathbb {Z}^2$, as

$$\begin{aligned} \widetilde{\beta }_{f,k}(p) := \bigg (\left\lvert \{\sigma \in K_k^+ \text { s.t. } f(\sigma ) \le p\}\right\rvert , \left\lvert \{\sigma \in K_{k+1}^- \text { s.t. } f(\sigma ) \le p\}\right\rvert \bigg ). \end{aligned}$$

The set of verbose Betti functions indexed by dimension is the *verbose Betti function (VBF)*, denoted $\widetilde{\beta }_f$. We drop subscripts when clear from context.

We emphasize that, just like VPDs, VBFs are *dimension-returning*, meaning we know how many simplices of a given dimension are added at a particular height. Further note that the VBF represents the BF as a parameterized count of positive and negative *k*-simplices, which means that the BF is known from the VBF, making the BF a weaker invariant than the VBF. Similarly, because the VBF is known from the VPD, we also have that the VBF is a weaker invariant than the VPD; the relationship between these and other descriptors is further explored in [15, 34].

***Euler Characteristic Functions***

The Euler characteristic of a simplicial complex $K$ is the alternating sum of the number of simplices of different dimensions: $\chi (K) = \sum _{i=0}^{{\dim (K)}} (-1)^i n_i$, where we recall that $n_i$ is the number of *i*-dimensional simplices in $K$. Given a filtration, the Euler characteristic with respect to the filtration parameter is known as the Euler characteristic function (ECF). Formally,

#### Definition 3

*(Euler Characteristic Function (ECF) and Verbose ECF)* Given a filter $f :K\rightarrow \mathbb {R}$, the *Euler characteristic function* $\chi _f: \mathbb {R}\rightarrow \mathbb {Z}$ is the step function:

5 $$\begin{aligned} \chi _{f}(p) := \sum _{k=0}^{\infty } (-1)^k n_k^{(p)}, \end{aligned}$$

where $n_k^{(p)}$ is the number of *k*-simplices in $f^{-1}(-\infty ,p]$.

Suppose $f'$ is an index filter compatible with *f*. Let $\sigma \in K$. If $\sigma$ appearing in the index filtration of $f'$ corresponds with the Euler characteristic increasing (respectively, decreasing), then $\sigma$ is even- (respectively, odd-) dimensional. The set of even (resp., odd) simplices is denoted by *E* (resp., *O*).

We define a verbose version of the ECF: the *verbose Euler characteristic function* (VECF) as the function $\widetilde{\chi }_f: \mathbb {R}\rightarrow \mathbb {Z}^2$, where

6 $$\begin{aligned} \widetilde{\chi }_{f}(p) := \bigg (\left\lvert \{\sigma \in E \text { s.t. } f(\sigma ) \le p\}\right\rvert , \left\lvert \{\sigma \in O \text { s.t. } f(\sigma ) \le p\}\right\rvert \bigg ). \end{aligned}$$

In other words, the VECF represents the ECF as a parameterized count of even- and odd-dimensional simplices.

We can read off the ECF from the PD by forgetting information, and the VECF from the VPD. In other words, the ECF is a weaker invariant than the PD and the VECF is a weaker invariant than the VPD.

***Directional Transforms***

Considering descriptors corresponding to lower-star filtrations from all possible directions, we obtain a *verbose directional transform*.

#### Definition 4

*(Directional Transforms)* Given a simplicial complex $K$ GP-immersed in $\mathbb {R}^d$ and a descriptor $\widetilde{\mathcal {A}}$, a *directional transform* of $K$ is the parameterized set of all directional descriptors of type $\widetilde{\mathcal {A}}$. This is denoted

7 $$\begin{aligned} \widetilde{\mathcal {A}}[K,\mathbb {S}^{d-1}] := \{(s,\widetilde{\mathcal {A}}_{h_s} )\}_{s\in \mathbb {S}^{d-1}}. \end{aligned}$$

If $\widetilde{\mathcal {A}}$ is verbose, we may specify that $\widetilde{\mathcal {A}}[K,\mathbb {S}^{d-1}]$ a *verbose directional transform*. Specifically, $\widetilde{\mathcal {D}}[K,\mathbb {S}^{d-1}]$ is the *verbose persistent homology transform (VPHT)*, $\widetilde{\beta }[K,\mathbb {S}^{d-1}]$ is the *verbose Betti transform (VBT)*, and $\widetilde{\chi }[K,\mathbb {S}^{d-1}]$ is the *verbose Euler characteristic transform (VECT)*.

Given a discrete set of directions, $S \subset \mathbb {S}^{d-1}$, we similarly define

8 $$\begin{aligned} \widetilde{\mathcal {A}}[K,S]:= \{(s,\widetilde{\mathcal {A}}_{h_s} )\}_{s\in S} \subset \widetilde{\mathcal {A}}[K,\mathbb {S}^{d-1}]. \end{aligned}$$

We call $\widetilde{\mathcal {A}}[K,S]$ a *discretization* of $\widetilde{\mathcal {A}}[K,\mathbb {S}^{d-1}]$. With *S* selected wisely, the hope is that $\widetilde{\mathcal {A}}[K,S]$ earries the same information as $\widetilde{\mathcal {A}}[K,\mathbb {S}^{d-1}]$.

#### Definition 5

*(Faithful Discretizations)* Given a simplicial complex $K$ GP-immersed in $\mathbb {R}^d$, a discrete set $S \subset \mathbb {S}^{d-1}$, and a descriptor type $\widetilde{\mathcal {A}}$, we say that $\widetilde{\mathcal {A}}[K,S]$ is a *faithful discretization of* $\widetilde{\mathcal {A}}[K,\mathbb {S}^{d-1}]$ if, for any simplicial complex $K' \ne K$ GP-immersed in $\mathbb {R}^d$, there exists $s\in S$ such that $\widetilde{\mathcal {A}}_{h_{s}}\ne \widetilde{\mathcal {A}}_{h_{s}'}$, where $h_s$ and $h'_s$ are lower-star filters of *K* and $K'$ with respect to direction *s*, respectively. That is, no other simplicial complex GP-immersed in $\mathbb {R}^d$ has the same parameterized set of directional descriptors.

### Tools for Building a Faithful Discretization

In this section, we introduce lemmas and tools that are used in our proofs. We begin with a lemma that connects simplices to points in a verbose diagram.

#### Lemma 6

(Simplex Count) Let $K$ be a simplicial complex, $k \in \mathbb {N}$, and $c \in \mathbb {R}$. Let $f :K\rightarrow \mathbb {R}$ be a filter function. Then, the *k*-dimensional simplices of $K$ with a function value of *c* are in one-to-one correspondence with the points in the following multiset:

9 $$\begin{aligned} \left\{ (a,b) \in \widetilde{\mathcal {D}}_{f, k} \text { s.t. } a = c \right\} \sqcup \left\{ (a,b) \in \widetilde{\mathcal {D}}_{f, k-1} \text { s.t. } b = c \right\} . \end{aligned}$$

The proof follows from definitions, and is included in Appendix C. Next, we define a structure that helps build a geometric intuition for several of the proofs that follow.

#### Definition 7

*(Filtration Hyperplane)* Let $s\in \mathbb {S}^{d-1}$ and let $c \in \mathbb {R}$. Let $H(s, c)$ be the $(d-1)$-dimensional hyperplane that passes through the point $cs\in \mathbb {R}^d$ and is perpendicular to $s$. We denote the closed half-space above (below) this hyperplane with respect to $s$ by $H^{\uparrow }(s, c)$ (respectively, $H^{\downarrow }(s, c)$).

Let *V* be a finite set of vertices in $\mathbb {R}^d$ and let $h_s :V \rightarrow \mathbb {R}$ be the lower-star filter function with respect to the direction *s*. The *filtration hyperplanes of* *V* are the set of hyperplanes $\mathbb {H}(s, V) := \{ H(s, h_s(v)) \}_{v \in V}$.

All hyperplanes in $\mathbb {H}(s, V)$ are parallel to each other and perpendicular to the direction $s$. For a simplicial complex *K* GP-immersed in $\mathbb {R}^d$, because a vertex will never cause a finite death, the births in $\widetilde{\mathcal {D}}_{h_s, 0}$ are in one-to-one correspondence with the vertices of *K* by Lemma 6. Thus, there is a filtration hyperplane at the height of each vertex. Furthermore, because events in a VPD, VBF, or VECF only occur at vertex heights, and each vertex height corresponds to at least one event, we know all filtration hyperplanes from any of these verbose descriptors.

By observing intersections of a sufficient number of linearly independent filtration hyperplanes, we form a grid of points of intersections:

#### Definition 8

*(Filtration Grid)* Let $S \subset \mathbb {S}^{d-1}$ be a finite set of directions such that $\textrm{span}\{S\} = \mathbb {R}^d$ and let $P\subset \mathbb {R}^d$ be a pointset. We define the *filtration grid of* *P* *with respect to* *S* to be the grid of points, *A*, arising from choosing one hyperplane $\mathbb {H}(s, P)$ for each $s \in S$. That is, the filtration grid is the collection of $|S |$-way intersections of filtration hyperplanes. Note that $|S |\ge d$, $P \subseteq A$, and $|A |\le |P |^d$.

Finally, we define the specific types of directions that are the building blocks of our faithful discretization. We begin with directions that are perpendicular to a simplex in a specific way.

#### Definition 9

*(P-Perpendicular)* Let $V \subset P \subset \mathbb {R}^d$ such that *P* is in general position. We say that a direction $s \in \mathbb {S}^{d-1}$ is *P*-*perpendicular* to *V* if for all $u \in P$ and $v \in V$, we have $s\cdot u = s\cdot v$ if and only if $u \in V$. In other words, $s$ is perpendicular to $\textrm{aff}(V)$ and no other vertex in *P* is at the same height as the vertices in *V* with respect to $s$.

The next definition describes when a direction is a slight tilt of an initial direction that is *P*-perpendicular to some *V*, so that a specified subset *W* (and no other points) pops above $V \setminus W$.

#### Definition 10

((*P*, *V*, *W*, *s*)-*Perturbation*) Let $W \subset V \subset P \subset \mathbb {R}^d$ such that *P* is in general position. Let $s\in \mathbb {S}^{d-1}$ be *P*-perpendicular to *V*. Then, we say a direction $s_* \in \mathbb {S}^{d-1}$ is a (*P*, *V*, *W*, *s*)-*perturbation* if the following hold:

1. The direction $s_*$ is *P*-perpendicular to $V\setminus W$.
2. The points in *W* are above $V \setminus W$ with respect to $s_*$.
3. For all $p \in P \setminus V$, *p* is strictly above (below) the height of $V \setminus W$ with respect to $s_*$ if and only if it is strictly above (below, respectively) *V* with respect to $s$.

If *V* or *W* define the vertices of a simplex $\sigma$, then we may use $\sigma$ in place of $\textrm{verts}(\sigma )$ in the definitions above.

## A Faithful Discretization

In this section, we give sufficient properties for a discretization of the VPHT, VBT, or VECT of a given simplicial complex to be faithful.

### Definition 11

*(Vertex-Isolating)* Given a simplicial complex *K* GP-immersed in $\mathbb {R}^d$ and given $S \subseteq \mathbb {S}^{d-1}$, we say that the pair (*K*, *S*) is *vertex-isolating* if *S* contains *d* linearly independent directions, denoted $\{s_1, s_2, \ldots , s_d\}$, and one additional direction $s$ such that the points in the filtration grid of $K_0$ with respect to $\{s_1, s_2, \ldots , s_d\}$ are uniquely ordered by their heights in direction *s*.

### Definition 12

*(Simplex-Isolating)* Let *K* be a simplicial complex GP-immersed in $\mathbb {R}^d$ and let $S \subseteq \mathbb {S}^{d-1}$. Let $\sigma$ be a simplex with $\textrm{verts}(\sigma ) \subseteq K_0$. We say that the pair (*K*, *S*) is $\sigma$-*isolating* if *S* includes a direction $s_{\sigma }$ such that:

1. $s_{\sigma }$ is $K_0$-perpendicular to $\sigma$, and
2. for each $\emptyset \ne W \subsetneq V=\textrm{verts}(\sigma )$, *S* includes a direction that is a $(K_0,V,W,s_{\sigma })$-perturbation.

**Fig. 1:**
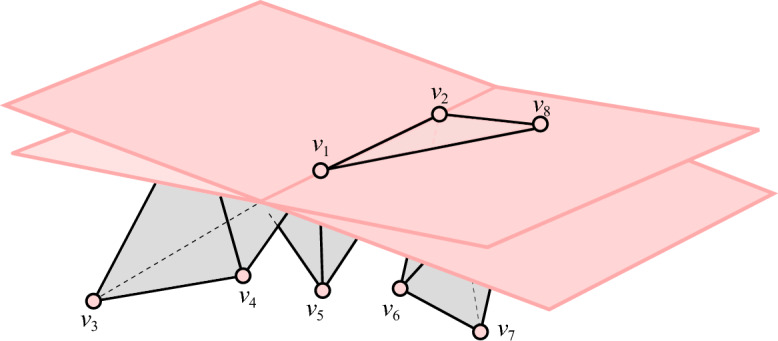
The filtration hyperplanes (shaded in pink) corresponding to a pair of $[v_1, v_2, v_8]$-isolating directions, where we have $V=\{v_1, v_2, v_8\}$ and $W= \{v_8\}$. One hyperplane corresponds to a direction $s_V$ that is $K_0$-perpendicular to $V = [v_1, v_2, v_8]$. The other corresponds to a direction that is a $(K_0, V, W, s_V)$-perturbation) which, by pivoting $s_V$ around $V\setminus W$, “pops” the vertex of *W* above the filtration hyperplane

If the pair (*K*, *S*) is $\sigma$-isolating for every simplex $\sigma \in K \setminus K_0$, we say that (*K*, *S*) is *simplex-isolating*. See Fig. 1.

Next, we make the observation that if there are directions that are $\sigma$-isolating for all maximal simplices of a complex, then the set of directions is simplex-isolating. Namely, this implies there are directions that are $\sigma$-isolating for *all* simplices of a complex, regardless if the simplices are maximal.

### Lemma 13

(Recursive Nature of $\sigma$-Isolating Directions) Let $K$ be a simplicial complex GP-immersed in $\mathbb {R}^d$, and let *S* be a set of directions such that (*K*, *S*) is $\sigma$-isolating for every maximal simplex $\sigma$. Then, (*K*, *S*) is $\tau$-isolating for every simplex $\tau \in K$. That is, (*K*, *S*) is simplex-isolating.

### Proof

Let $\tau \in K$; we aim to show (*K*, *S*) is $\tau$-isolating. Either $\tau$ is a maximal simplex or $\tau$ is a proper face of a maximal simplex. If $\tau$ is maximal, the claim follows immediately by assumption. Suppose, then, that $\tau$ is a proper face of a maximal simplex $\tau _m$. Denote the vertex sets of $\tau$ and $\tau _m$ by *T* and $T_m$, respectively.

First, we show *S* contains a direction that is $K_0$-perpendicular to $\tau$. Because *S* satisfies Statement (1) of Definition 12, there is some $s_m \in S$ that is $K_0$-perpendicular to $T_m$. Then, because *S* also satisfies Statement (2), and, in particular, when $V=T_m$ and $W=T_m \setminus T$, there is a direction $s_\tau \in S$ that is a $(K_0, T_m, T_m \setminus ~T, s_m)$-perturbation; $s_\tau$ is $K_0$-perpendicular to $T_m \setminus (T_m \setminus T) = T$, as desired.

Next, we show that *S* contains a direction that is a $(K_0, \tau , \tau ', s_\tau )$-perturbation for every $\tau '\prec \tau$. If $\tau$ is a vertex, the claim is vacuously true. We therefore proceed assuming there exists some $\tau ' \prec \tau$ and let $T' = \textrm{verts}(\tau ')$. Because $T_m \setminus (T \setminus T') \subset T_m$, and because *S* satisfies Statement (2) of Definition 12, there exists a direction $s_p \in S$ that is a $(K_0, T_m, T_m \setminus (T \setminus T'), s_m)$-perturbation. We show that $s_p$ satisfies the three properties of Definition 10. By definition, $s_p$ is $K_0$-perpendicular to $T_m \setminus (T_m \setminus (T \setminus T') = T \setminus T'$. Hence, $s_p$ satisfies Statement (1) of Definition 10. Also by definition, the points of $T_m \setminus (T \setminus T')$ are above $T \setminus T'$ with respect to $s_p$. Because $T' \subset T_m \setminus (T \setminus T')$, we conclude that the points in $T'$ are above $T \setminus T'$ with respect to $s_p$. Hence, $s_p$ satisfies Statement (2) of Definition 10. Finally, let $p \in K_0 \setminus T$. If $p \in K_0 \setminus T_m$, then *p* is strictly above (below) the height of $\tau \setminus \tau '$ with respect to $s_p$ if and only if it is strictly above (below) the height of $\tau \setminus \tau '$ with respect to $s_m$ by definition. Furthermore, this means *p* is above (below, respectively) $\tau \setminus \tau '$ if and only if it is above (below) with respect to $s_\tau$. If, instead, $p \in K_0 \setminus (T_m \setminus T)$, then $p \in \textrm{verts}(\sigma ) \setminus (T \setminus T')$, so *p* is necessarily above $\tau \setminus \tau '$ with respect to $s_\tau$ and $s_p$. Hence, $s_p$ satisfies Statement (3) of Definition 10 and $s_p$ is a $(K_0, \tau , \tau ', s_\tau )$-perturbation, as desired. $\square$

We arrive at our first main theorem and related corollary:

### Theorem 14

(Sufficient Conditions for Faithful Discretization) Let $K$ be a simplicial complex GP-immersed in $\mathbb {R}^d$ such that $\dim (K)< d$ and let $S \subset \mathbb {S}^{d-1}$ be a discrete set such that $(K,S)$ is vertex- and simplex-isolating. Let $\widetilde{\mathcal {A}}\in \{\widetilde{\mathcal {D}}, \widetilde{\beta }, \widetilde{\chi }\}$. Then, $\widetilde{\mathcal {A}}[K,S]$ is a faithful discretization of $\widetilde{\mathcal {A}}[K,\mathbb {S}^{d-1}]$.

### Corollary 15

(Subcomplexes are Represented) Let $K$ be a simplicial complex GP-immersed in $\mathbb {R}^d$, let $S \subset \mathbb {S}^{d-1}$ satisfy the assumptions of Theorem 14, and let *L* be any subcomplex of *K*. Let $\widetilde{\mathcal {A}}\in \{\widetilde{\mathcal {D}}, \widetilde{\beta }, \widetilde{\chi }\}$. Then $\widetilde{\mathcal {A}}[K,S]$ is a faithful discretization of $\widetilde{\mathcal {A}}[L,\mathbb {S}^{d-1}]$.

The following remark emphasizes that the use of verbose descriptors, rather than concise descriptors, is vital to our results.

### Remark 16

*(Verbose Advantages)* Already, we see the dramatic advantages of using verbose descriptors over concise (non-verbose) descriptors. For example, known bounds for concise discretizations, e.g., given in [11], are stated for restricted classes of simplicial complexes. Changing these restrictions slightly leads us to constructions where this bound on the number of concise descriptors in a faithful representation is arbitrarily large, but the number of verbose descriptors described in Theorem 14 remains constant (and small); see Appendix A for further details.

## Reconstruction-Based Proof of Theorem 14

In this section, we prove Theorem 14 (Sufficient Conditions for Faithful Discretization) by using the discretization to reconstruct a simplicial complex. Our method first finds all zero-simplices, then all one-simplices, and so on. We begin by framing all arguments with respect to the VPHT and discuss extensions to the VBT and VECT in later sections.

### Vertex Reconstruction

Vertex reconstruction using a vertex-isolating set is an established result.

#### Lemma 17

(Vertex Reconstruction, [3, Theorem 9]) Let *K* be a simplicial complex GP-immersed in $\mathbb {R}^d$. Then, given a set of directions *S* that is vertex-isolating, $\widetilde{\mathcal {D}}[K,S]$ can reconstruct $K_0$.

**Fig. 2:**
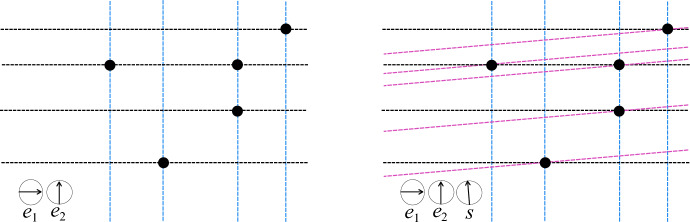
The vertex set *P* (large black points) defines the filtration grid of *P* with respect to $\{e_1, e_2\}$, denoted *A* (grey and black dots in the left figure). Because $e_1$ and $e_2$ are linearly independent and because the direction *s* uniquely orders the points of the *A*, the set $\{e_1, e_2, s\}$ is vertex-isolating for the given vertex set. To locate the vertices of the set, we simply need to identify all intersections of $\mathbb {H}(s, P)$ (tilted pink dashed lines) with *A*

We refer the reader to [3] for details; simplex-isolating directions create exactly $n_0$ $(d+1)$-way intersections of their filtration hyperplanes. It is then a simple matter of checking for the locations of these intersections. See Fig. 2.

### Higher-Dimensional Simplex Reconstruction

In this section, we focus on methods for finding higher-dimensional simplices, assuming that zero-dimensional simplices have already been found. The key to determining whether a simplex exists is the *k*-*indegree* of a potential simplex, $\sigma$, which is the count of *k*-dimensional cofaces of $\sigma$ contained in *K* occurring at the same height as $\sigma$ in a particular direction. Importantly, these cofaces need not be proper, so for the particular case when $k = \dim (\sigma )$, we count 1 if $\sigma \in K$, and 0 if $\sigma \not \in K$.

#### Definition 18

*(k*-*Indegree for Simplex)* Let $K$ be a simplicial complex GP-immersed in $\mathbb {R}^d$ and let $\sigma \subset \mathbb {R}^d$ be a simplex such that $\textrm{verts}(\sigma ) \subseteq K_0$. Furthermore, let $s\in \mathbb {S}^{d-1}$ be a direction $K_0$-perpendicular to $\sigma$. Then, the *k*-*indegree of* $\sigma$ *in direction* $s$ is the number of *k*-dimensional cofaces of $\sigma$ contained in *K* that have the same height as $\sigma$ in direction $s$. We say that such a coface *contributes* to the *k*-indegree of $\sigma$ in direction $s$.

**Fig. 3:**
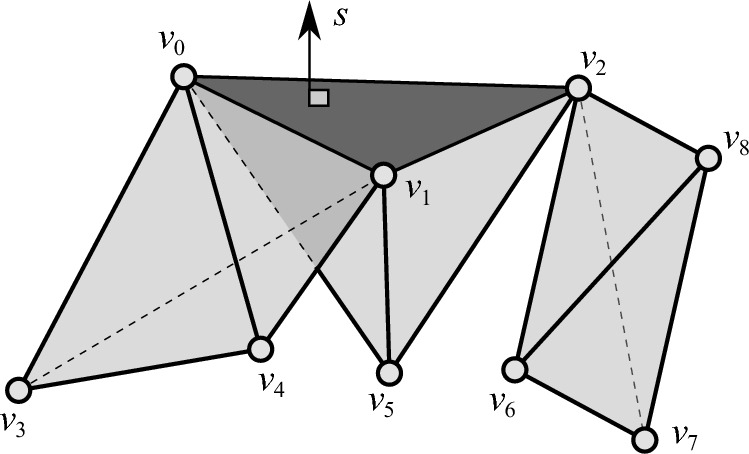
Computing the three-indegree for a two-simplex (triangle) in $\mathbb {R}^4$. The direction $s\in \mathbb {S}^3$ is orthogonal to $\textrm{aff}(\sigma )$, where $\sigma =[v_0,v_1,v_2]$ (shown in dark gray). The three vertices $v_0$, $v_1$, and $v_2$ are at the same height with respect to $s$, and all other vertices shown are below $\sigma$ (note that $s=s_{\sigma }$ from Definition 12(1)). Although the three-indegree of $\sigma$ is one, the VPD in direction $s$ sees three tetrahedron at the same height as $\sigma$. We use the three-indegree of all faces of $\sigma$ in tilted directions (that is, directions given in Definition 12(2)). Here, $[v_0, v_1]$ and $[v_2]$ are the only faces with non-zero three-indegree in the tilted directions; they both have indegree values of one. Using Corollary 20, we subtract these values from the number of three-simplices at height $s\cdot \sigma$. Then, we find $3-1-1=1$, the correct value for the three-indegree of $\sigma$ in direction $s$

If a direction $s$ is $K_0$-perpendicular to $\textrm{aff}(\sigma )$, all zero-simplices of $\sigma$ are at the same height in direction $s$. However, not all *k*-simplices at this height contribute to the *k*-indegree of $\sigma$, as shown in Fig. 3. Thus, if we only use diagrams that are $K_0$-perpendicular to a simplex, we may overcount *k*-indegree. To correctly compute *k*-indegree, we combine information from $\sigma$-isolating directions (Definition 12), using an inclusion-exclusion type formula. Directions that are $\sigma$-isolating are slight perturbations of *s*, isolating faces of $\sigma$, so that we can check for *k*-simplices at the height of $\sigma$ that are “attached” to $\sigma$, but that are not cofaces of $\sigma$.

**Algorithm 1:**
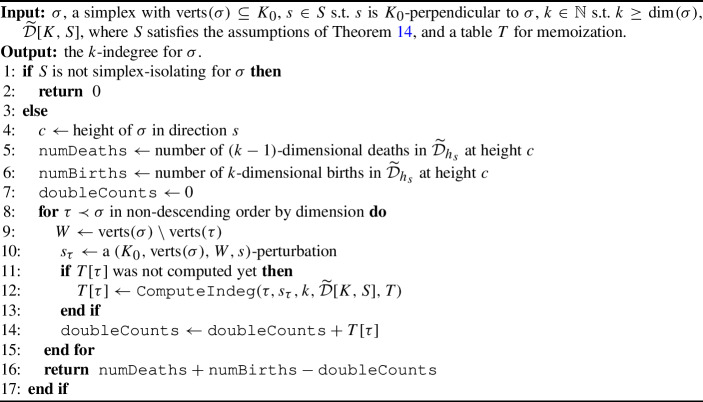
$$\mathtt{{ComputeIndeg}}(\sigma , s, k, \widetilde{\mathcal {D}}[K,S], T = \{ \} )$$

To prove the correctness of Algorithm 1, we make the following observation.

#### Lemma 19

(Indegree Contributors) Let $K$ be a simplicial complex GP-immersed in $\mathbb {R}^d$. Let $\tau$ and $\sigma$ be potential simplices with $\textrm{verts}(\tau ) \subset \textrm{verts}(\sigma ) \subseteq K_0$, and let $\sigma '\in K$. Let *k* denote $\dim (\sigma ')$. Let $s$ be $K_0$-perpendicular to $\sigma$ and let $s_\tau$ be a $(K_0, \sigma , \sigma \setminus \tau , s)$-perturbation. Then, $\sigma '$ contributes to the *k*-indegree of $\tau$ in direction $s_\tau$ if and only if $\sigma '$ is at the same height as $\sigma$ in direction $s$ and $\tau = \sigma \cap \sigma '$.

#### Proof

Let $f,f_{\tau } :K\rightarrow \mathbb {R}$ be the filter function for direction $s$ and $s_\tau$, respectively.

($\Rightarrow$) Suppose that $\sigma '$ contributes to the *k*-indegree of $\tau$ in direction $s_\tau$. Then, by the definition of *k*-indegree, $\tau \preceq \sigma '$ and $\sigma '$ is at the same height as $\tau$ with respect to direction $s_\tau$. Because $s_\tau$ is a $(K_0, \sigma , \sigma \setminus \tau , s)$-perturbation, this means $\sigma '$ is at the same height as $\tau$ in direction *s*, and therefore also at the same height as $\sigma$ in direction $s$. Because $\tau \prec \sigma$ by assumption, we have $\tau \preceq \sigma \cap \sigma '$. We must now show that $\sigma \cap \sigma ' \preceq \tau$.

By contradiction, suppose that $\sigma \cap \sigma ' \npreceq \tau$. Then, there exists a vertex $v \in \sigma \cap \sigma '$ such that $v \notin \tau$. Thus, $v \in \sigma \setminus {\tau }$. Because $s_{\tau }$ is a $(K_0, \sigma , \sigma \setminus \tau , s)$-perturbation, we know that $s_\tau \cdot v > s_\tau \cdot w$ for all $w \in \textrm{verts}(\tau )$, a contradiction to the claim that $\sigma '$ contributes to the *k*-indegree of $\tau$ in direction $s_\tau$. Therefore, $\sigma \cap \sigma ' \preceq \tau$ as required.

($\Leftarrow$) Suppose that $\sigma '$ is at the same height as $\sigma$ in direction $s$ and that $\tau = \sigma \cap \sigma '$. If $\tau = \sigma '$, the claim follows from the definition of *k*-indegree. Then, suppose $\tau \prec \sigma '$. Denote the dimension of $\tau$ by *j*. Because $\sigma '$ is a *k*-simplex, $\tau$ is a *j*-simplex, and $\tau \prec \sigma '$, we can write $\tau = [v_0, v_1, \ldots , v_j]$ and $\sigma ' = [v_0,v_1,\ldots , v_k]$ where $v_i \in K_0$. Then,

10 $$\begin{aligned} f_\tau (\sigma ') = \max _{i=0}^k f_\tau (v_i)= &   \max \left( \max _{i=0}^j f_\tau (v_i) , \max _{i=j+1}^k f_\tau (v_i) \right) \nonumber \\= &   \max \left( f_\tau (\tau ), \max _{i=j+1}^k f_\tau (v_i) \right) . \end{aligned}$$

Because $\sigma '$ is at the same height as $\sigma$ in direction $s$ and $\tau \prec \sigma$, $\sigma '$ is also at the same height as $\tau$ in direction $s$, meaning that $f(v_i) \le f(\tau )$ for all $0 \le i \le k$. Because $v_i$ is not in $\tau$ for $i > j$, it must also be the case that $f(v_i) < f(\tau )$ for $i > j$. Because $s_\tau$ is a $(K_0, \sigma , \sigma \setminus \tau , s)$-perturbation, by Statement (3) of Definition 10, any vertex below $\tau$ in direction $s$ is also below $\tau$ in direction $s_\tau$. Thus, $f_\tau (v_i) < f_\tau (\tau )$ for all $j < i \le k$ and

11 $$\begin{aligned} \left( \max _{i=j+1}^k f_\tau (v_i) \right) < f_\tau (\tau ). \end{aligned}$$

Then, by Equation (10), $f_\tau (\sigma ')=f_\tau (\tau )$. This taken together with $\tau \prec \sigma '$ shows that $\sigma '$ contributes to the *k*-indegree of $\tau$ in direction $s_\tau$. $\square$

We make note of three points. First, the set of *k*-simplices at the same height as $\sigma$ is a superset of the set of *k*-simplices that *contribute* to the *k*-indegree of $\sigma$ in this direction, i.e., those that additionally have $\sigma$ as a face. Second, the contributors to the *k*-indegree of $\sigma$ also contribute to the *k*-indegree of proper faces $\tau$ of $\sigma$, unless we consider the *k*-indegree of $\tau$ in a tilted direction that is specifically $K_0$-perpendicular to $\tau$ but not to $\sigma$. And finally, if a *k*-simplex is at the same height as $\sigma$ but does not contribute to the *k*-indegree of $\sigma$, it contributes to some proper face of $\sigma$ instead. Thus, in order to ensure we only count the contributors for $\sigma$, our inclusion-exclusion type calculation of Algorithm 1 isolates each proper face $\tau$ of $\sigma$ and computes the *k*-indegree of $\tau$ with respect to tilted directions (Line 10). By recursively computing the *k*-indegree of faces of $\sigma$ in tilted directions, we add or subtract the number of *k*-simplices at the same height as $\sigma$, alternating by dimension. This ensures that no coface of $\tau$ that is not a coface of $\sigma$ adds to the *k*-indegree of $\sigma$. Lemma 19 identifies which simplices contribute to the *k*-indegree of $\sigma$, as well as which simplices do not and, instead, contribute to the *k*-indegree of some proper face of $\sigma$. Following the positive and negative effects of each simplex, the calculation telescopes back to correcting the total count simply by subtracting off any counted *k*-simplices that do not have $\sigma$ as a face; see Fig. 3. This calculation is summarized in the following corollary.

#### Corollary 20

Let $K$ be a simplicial complex GP-immersed in $\mathbb {R}^d$, let $\sigma$ be a potential simplex with $\textrm{verts}(\sigma ) \subseteq K$, and *s* a direction in $\mathbb {S}^{d-1}$ that is $K_0$-perpendicular to $\sigma$. For $\tau \prec \sigma$, let $s_\tau$ denote a $(K_0, {\sigma }, {\sigma \setminus \tau }, s)$-perturbation, and let $\delta _\tau$ denote the *k*-indegree of $\tau$ in direction $s_\tau$ for some $k \ge \dim (\sigma )$. Finally, let $\delta$ denote the number of *k*-simplices born at the height of $\sigma$ with respect to direction *s*. Then, the *k*-indegree of $\sigma$ is

12 $$\begin{aligned} \delta - \sum _{\tau \prec \sigma } \delta _{\tau }. \end{aligned}$$

We prove the correctness of Algorithm 1 in the following theorem.

#### Theorem 21

(Computing *k*-Indegree) Let $K$ be a simplicial complex GP-immersed in $\mathbb {R}^d$. Let $\sigma$ be a potential simplex with $\textrm{verts}(\sigma ) \subseteq K_0$ and $s\in \mathbb {S}^{d-1}$ such that $s$ is $K_0$-perpendicular to $\sigma$. Then, for $k \ge \dim (\sigma )$, $\mathtt{{ComputeIndeg}}(\sigma , s,k,\widetilde{\mathcal {D}}[K,S])$ returns the *k*-indegree of $\sigma$ in direction $s$.

#### Proof

First, we note that because *S* is assumed to be simplex-isolating for *K*, if *S* is not simplex-isolating for $\sigma$, then $\sigma$ is not a simplex of *K* and thus must have *k*-indegree zero, which is the value returned on Line 2.

Suppose then that *S* is simplex-isolating for $\sigma$. We prove the claim inductively on $j= \dim (\sigma )$. For the base case $j=0$, consider the zero-simplex [*v*]. Let $h_s:K\rightarrow \mathbb {R}$ be the filter function for direction $s$. We note that this base case is a generalization of [3, Lemma 11]. However, unlike in [3, Lemma 11], we are only making an argument for the *k*-indegree at a single vertex and not all vertices. As such, we can relax the requirement that no two vertices in $K_0$ have the same height in direction $s$ and just require that no vertices in $K_0 \setminus \{v\}$ have the same height in direction $s$ as *v*. Thus, we have that *k*-indegree of $\sigma$ is equal to the number of *k*-simplices that have height $h_s(v)$, which, by Lemma 6, is:

13 $$\begin{aligned} |f^{-1}(f(v))|= |\{ (a,b) \in \widetilde{\mathcal {D}}_{h_s, k-1} \text { s.t. } b = f(v) \} |+ |\{ (a,b) \in \widetilde{\mathcal {D}}_{h_s, k} \text { s.t. } a = f(v) \} |. \end{aligned}$$

In Algorithm 1, notice that if $\sigma$ is a single vertex, we do not enter the loop that starts on Line 8. Thus, the return value is exactly the number given in Equation (13).

For the inductive assumption, let $j \ge 0$. We assume that Algorithm 1 returns the $k'$-indegree of $\tau$ in direction $s$, for all $\tau \in K_j$ and all $k'\ge j$.

For the inductive step, let $\dim (\sigma ) = j+1$. Let $k \ge j+1$. Now, we compute the *k*-indegree of $\sigma$ in direction $s$. Using Lemma 6, we know that the number of *k*-simplices with height $h_s(\sigma )$ in direction $s$ is:

14 $$\begin{aligned} \delta := |\{ (a,b) \in \widetilde{\mathcal {D}}_{h_s, k-1} \text { s.t. } b = f(\sigma ) \} |+ |\{ (a,b) \in \widetilde{\mathcal {D}}_{h_s, k} \text { s.t. } a = f(\sigma ) \} |. \end{aligned}$$

Let $F_\sigma$ denote this set of simplices, let $\sigma ' \in F_{\sigma }$, and let $\tau \prec \sigma$. Suppose that $s_\tau$ is a $(K_0, \textrm{verts}(\sigma ), \textrm{verts}(\sigma \setminus \tau ), s)$-perturbation. By Lemma 19, the *k*-simplex $\sigma '$ contributes to the *k*-indegree of $\tau$ in direction $s_\tau$ if and only if $\tau = \sigma \cap \sigma '$.

From Equation (14) we get $\delta$, the number of *k*-simplices born at the height of $\sigma$. In Algorithm 1, numDeaths+numBirths is equal to $\delta$ in Equation (12), and the values $\delta _{\tau }$ are computed in Line 12 of Algorithm 1. Thus, the return value matches Equation (12), and, by Corollary 20, equals the *k*-indegree of $\sigma$. $\square$

### Proof of Theorem 14 for the VPHT

Using the results from the previous subsection, we arrive at Algorithm 2 that fully reconstructs a GP-immersed simplicial complex. Central to this process is using *k*-indegree to certify the presence or absence of a simplex.

**Algorithm 2:**
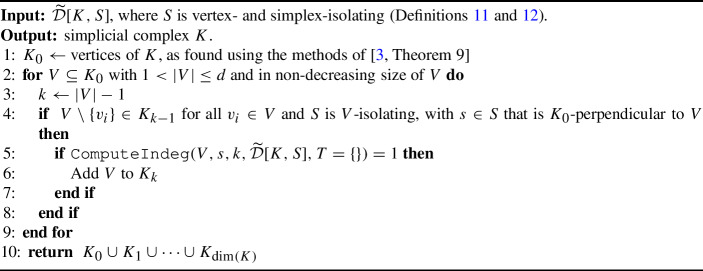
$$\mathtt{{ReconstructComplex}}(\widetilde{\mathcal {D}}[K,S])$$

#### Theorem 22

(Simplicial Complex Reconstruction) Let $K$ be a ${\dim (K)}$-dimensional simplicial complex GP-immersed in $\mathbb {R}^d$, such that ${\dim (K)}\le d-1$. Let $S \subset \mathbb {S}^{d-1}$ satisfy the assumptions of Theorem 14 (that is, $(K,S)$ is vertex- and simplex-isolating). Then, Algorithm 2 reconstructs $K$, so $\widetilde{\mathcal {D}}[K,S]$ is a faithful discretization of $\widetilde{\mathcal {D}}[K,\mathbb {S}^{d-1}]$.

#### Proof

We begin by reconstructing $K_0$ on Line 1 using the methods of [3, Theorem 9]. Algorithm 2 then iterates over all subsets of vertices $V \subseteq K_0$. We do not yet know if the simplex defined by *V* is in *K*. Because sets are included in non-decreasing size, $K_{k-1}$ is finalized by the time *V* is considered. The condition that the boundary of the simplex defined by *V* is contained in $K_{k-1}$ is checked on Line 4. Because $\widetilde{\mathcal {D}}[K,S]$ is simplex-isolating, if *V* defines a *k*-simplex of *K*, the set *S* will contain a direction *s* that is $K_0$-perpendicular to *V*. Thus, if there is no such direction, we know *V* does not define a simplex of *K*. If there is such a direction, by Theorem 21, $\mathtt{{ComputeIndeg}}(V, s, k, \widetilde{\mathcal {D}}[K,S], T = \{ \} )$ (Algorithm 1) returns the number of *k*-simplices at the height of $\textrm{aff}(V)$ that contain the simplex defined by *V* as a face; because $k = |V |-1$, this is either zero if *V* does not define a *k*-simplex of *K*, or one if *V* does define a *k*-simplex of *K*. In the latter case, we add *V* to $K_k$. Because we iterate over all subsets of $K_0$, the algorithm eventually finds all simplices.

Finally, because we have shown $\mathtt{{ReconstructComplex}}(\widetilde{\mathcal {D}}[K,S])$ reconstructs *K*, we know $\widetilde{\mathcal {D}}[K,S]$ is a faithful discretization of $\widetilde{\mathcal {D}}[K,\mathbb {S}^{d-1}]$. $\square$

This theorem concludes the proof of Theorem 14 for the VPHT.

### Proof of Theorem 14 for the VBT

While Algorithm 2 and the algorithms referenced therein are phrased for verbose persistence diagrams, the algorithms only ever use the dimension and heights of simplices in a given direction.^2^ That is, they never make use of the birth-death pairing information unique to persistence diagrams.

Superficial adaptations to the algorithms of Section 4 give us a reconstruction method for when the necessary data of the heights and dimensions of simplices instead comes to us from a verbose Betti function. Thus, the same set of directions that faithfully discretize the VPHT faithfully discretize the VBT, so we see that Theorem 14 also holds for the VBT.

### Proof of Theorem 14 for the VECT

Unlike $\text {VPDs}$ or $\text {VBFs}$, $\text {VECFs}$ do not contain information about the dimension of each simplex, so we no longer can directly compute *k*-indegree. However, we are still able to use a set of directions that is vertex- and simplex-isolating to reconstruct all simplices. The remainder of this section serves to prove this claim. Our main tool is an adaptation of the definition of *k*-indegree to even/odd-indegree, where, instead of counting simplices of dimension *k*, we count even- (or odd-) dimensional simplices.

#### Definition 23

*(Even/Odd-Indegree for Simplex)* Let $K$ be a simplicial complex GP-immersed in $\mathbb {R}^d$ and let $\sigma \subset \mathbb {R}^d$ be a simplex such that $\textrm{verts}(\sigma ) \subseteq K_0$. Let $s\in \mathbb {S}^{d-1}$ be a direction $K_0$-perpendicular to $\textrm{aff}(\sigma )$. Then, the *even-indegree of* $\sigma$ *in direction* $s$ (respectively, *odd-indegree* of $\sigma$ in direction $s$) is the number of even-dimensional (respectively, odd-dimensional) cofaces of $\sigma$ contained in *K* that have the same height as $\sigma$ with respect to the direction $s$. We say that such a coface *contributes* to the even- (odd-)indegree of $\sigma$ in direction $s$.

Just as we did with the notion of *k*-indegree, we keep track of how many times we double-count faces of a simplex in order to correctly find the even/odd-indegree of that simplex. The following lemma helps us prove the correctness of this calculation.

#### Lemma 24

(Even/Odd-Indegree Contributors) Let $K$ be a simplicial complex GP-immersed in $\mathbb {R}^d$. Let $\tau$ and $\sigma$ be potential simplices with $\textrm{verts}(\tau ) \subset \textrm{verts}(\sigma ) \subseteq K_0$ and let $\sigma '\in K$. Suppose $\dim (\sigma ')$ is even (respectively, odd). Let $s$ be a direction that is $K_0$-perpendicular to $\sigma$ and let $s_\tau$ be a direction that is a $(K_0, \sigma , \sigma \setminus \tau , s)$-perturbation. Then, $\sigma '$ contributes to the even-indegree (respectively, odd-indegree) of $\tau$ in direction $s_\tau$ if and only if $\sigma '$ is at the same height as $\sigma$ in direction $s$ and $\tau = \sigma \cap \sigma '$.

The proof is identical to that of Lemma 19, and we have a nearly identical version of Corollary 20. We now present Algorithm 3, which computes even-indegree. The case for computing odd-indegree is nearly identical. We assert the correctness of Algorithm 3 and its odd counterpart in the following theorem.

**Algorithm 3:**
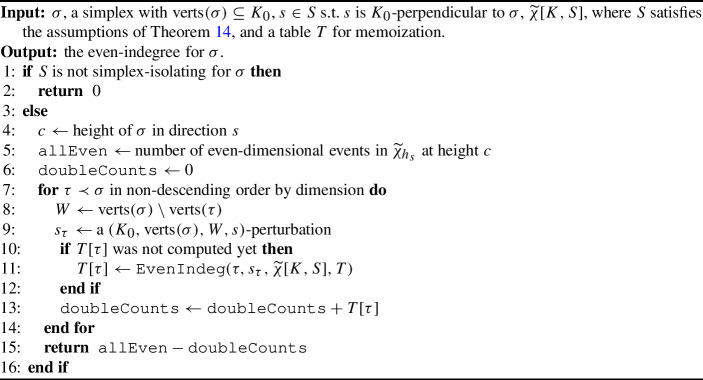
$$\mathtt{{EvenIndeg}}(\sigma , s, \widetilde{\chi }[K,S], T = \{ \} )$$

#### Theorem 25

(Computing Even/Odd-Indegree) Let $K$ be a simplicial complex GP-immersed in $\mathbb {R}^d$. Let $\sigma$ be a simplex with $\textrm{verts}(\sigma ) \subseteq K_0$ and $s\in \mathbb {S}^{d-1}$ such that $s$ is $K_0$-perpendicular to $\sigma$. Then, for $k \ge \dim (\sigma )$, $\mathtt{{EvenIndeg}}(\sigma , s,\widetilde{\chi }[K,S])$ returns the even-indegree of $\sigma$ in direction $s$. Similarly, the odd-version of this algorithm, $\mathtt{{OddIndeg}}(\sigma , s,\widetilde{\chi }[K,S])$ returns the odd-indegree of $\sigma$ in direction $s$.

We provide the proof in Appendix D.1, as it is nearly identical to the proof of Theorem 21, the parallel statement for *k*-indegree and $\text {VPDs}$.

The last step of the full process of reconstruction is to note that vertex-reconstruction only relied on the presence of filtration hyperplanes, arising from vertex-isolating directions. Because any verbose descriptor contains complete information about filtration hyperplanes, we can use VECFs to reconstruct $K_0$. Finally, we give a reconstruction algorithm and state the following main result. Similar to how Algorithm 2 uses *k*-indegree to certify the presence or absence of a *k*-simplex, Algorithm 4 uses even/odd-indegree to certify the presence or absence of a *k*-simplex.

**Algorithm 4:**
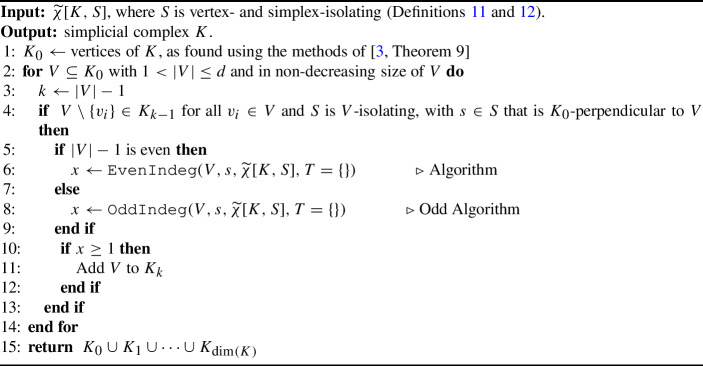
$$\texttt {VECFReconstructComplex}(\widetilde{\chi }[K, S])$$

#### Theorem 26

(Sufficient Conditions for Faithful Discretization of the VECT) Let $K$ be a simplicial complex GP-immersed in $\mathbb {R}^d$ such that $\dim (K) < d$ and let $S \subset \mathbb {S}^{d-1}$ such that $(K,S)$ is vertex- and simplex-isolating. Then, Algorithm 4 reconstructs *K*, so $\widetilde{\chi }[K,S]$ is a faithful discretization of $\widetilde{\chi }[K,\mathbb {S}^{d-1}]$.

Again, as the proof is nearly identical to the reasoning given in the proof of Theorem 22, we relegate the proof to Appendix D.1. This theorem concludes the proof of Theorem 14 for the VECT.

## Explicitly Building a Faithful Set

In this section, given a simplicial complex $K$ GP-immersed in $\mathbb {R}^d$, we provide algorithms to explicitly construct a set of directions $S \subseteq \mathbb {S}^{d-1}$ so that (*K*, *S*) is vertex- and simplex-isolating; see Appendix Appendix E for an example of the algorithms applied to a specific simplicial complex.

### Auxiliary Constructions

First, we describe the auxiliary methods used to compute vertex- and simplex-isolating directions.

***Computing a Perpendicular Direction***

We often compute directions orthogonal to the affine plane spanned by some set of $k+1$ points. To do so, we use Gram–Schmidt orthogonalization to first find *k* vectors in the space spanned by the points, and then again perform Gram–Schmidt orthogonalization on the standard basis vectors until we find one not in the space spanned by the points. While intuitive, an explicit formulation is not easily accessible from the literature, so we provide Algorithm 8 in Appendix D.2. As a consequence of this algorithm, we get the following lemma.

#### Lemma 27

(Computing a Perpendicular Direction) Let $P \subset \mathbb {R}^d$ be a point set in general position with $|P |\le d$. Then, we can compute a direction $s \in \mathbb {S}^{d-1}$ orthogonal to $\textrm{aff}(P)$ and in $\Theta (d^3)$ time.

***Plane Filling***

Given a point set $P \subset \mathbb {R}^d$ of $k+1\le d$ affinely independent points and a direction $s$ perpendicular to $\textrm{aff}(P)$, we want to find enough additional points in $\mathbb {R}^d$ so that they, along with the original point set *P*, “fill” the plane orthogonal to $s$. We provide pseudocode for this procedure and details of its correctness in Appendix D.2. A consequence of Algorithm 9 is the following lemma.

#### Lemma 28

(Plane Filling) Let $P \subset \mathbb {R}^d$ be a point set in general position with $|P |\le d$. Given a direction $s$ perpendicular to $\textrm{aff}(P)$, we can compute a complementary set of points $P'$ such that $\textrm{aff}(P' \cup P)$ has only two perpendicular directions, $s$ and $-s$ in $\Theta (d^3)$ time.

***Tilting***

In the Algorithm 5, we find a direction that is a slight tilt of one input direction towards another, so that no vertex orders change. To help explain the geometric intuition of the algorithm, let *S* be the set of *n* line segments in $\mathbb {R}^2$:

15 $$\begin{aligned} S := \bigg \{ \overline{(0,s\cdot p), (1,s' \cdot p)} \bigg \}_{p \in P}. \end{aligned}$$

**Fig. 4:**
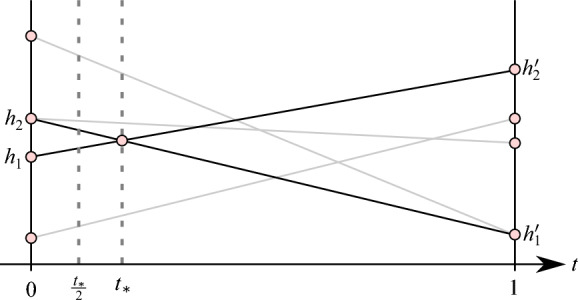
The solid grey lines in the figure above indicate the changing heights of points as we swing direction $s$ towards $s'$. Although we do not explicitly compute the grey lines, we know by simple geometry that no intersection of grey lines (and, in particular, no swapping of point orders) occurs before the *t*-value $t_*$, which corresponds to the intersection of the closest pairwise heights of points on the left and the extremal heights of points on the right, as indicated by the black lines. Because there are no crossings of line segments before $\frac{1}{2}t_*$, there is therefore no change in the order of points with respect to direction $s_t = (1-\frac{1}{2}t_*)s+ \frac{1}{2}t_* s'$

Each line segment in *S* represents a linear interpolation between the points $(0,s\cdot p)$ and $(1, s' \cdot p)$, which correspond to the heights in directions $s$ and $s'$ of each point in *P*. We parameterize each line segment in *S* as $(1-t)s\cdot p + t s' \cdot p$ for $t \in [0,1]$; see the grey and black solid lines in Fig. 4. Then, the vertical cross sections record point heights with respect to some direction that is an interpolation of $s$ and $s'$. We want to identify some $t_* > 0$ such that the ordering of the heights of points in direction $s_t = (1-t_*)s+ t_* s'$ is consistent with the ordering of the points in direction $s$. Notice that no swapping of point order can occur before the intersection of black lines in Fig. 4; thus, we choose the direction that is halfway to this point of the interpolation (labeled as $t_*/2$ in Fig. 4) as the output of Algorithm 5.

**Algorithm 5:**
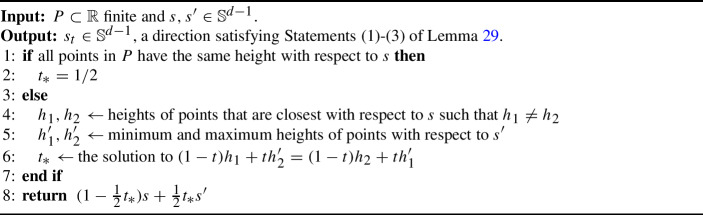
$$\mathtt{{Tilt}}(s,s',P)$$

#### Lemma 29

(Tilt) Let $P \subset \mathbb {R}^d$ be a finite set. Let $s, s' \in \mathbb {S}^{d-1}$. Then, using Algorithm 5, we can compute a direction $s_t=\mathtt{{Tilt}}(s,s',P)$ in $\Theta (|P |\log |P |+ d)$ time such that the following properties holds for all points $p_1,p_2 \in P$:

1. If $p_1$ is strictly above (below) $p_2$ with respect to direction $s$, then $p_1$ is strictly above (below, respectively) $p_2$ with respect to direction $s_t$.
2. If $p_1$ and $p_2$ are at the same height with respect to direction $s$ and $p_1$ is strictly above (below) $p_2$ with respect to direction $s'$, then $p_1$ is strictly above (respectively, below) $p_2$ with respect to direction $s_t$.
3. If $p_1$ is at the same height as $p_2$ with respect to both directions $s$ and $s'$, then $p_1$ and $p_2$ are at the same height with respect to direction $s_t$.

#### Proof

First, suppose all points in *P* have the same height with respect to $s$, satisfying the if statement on Line 1. Statement (1) is trivially satisfied. Consider the lines connecting the height $s \cdot P$ to the heights of points with respect to $s'$. There are no internal crossings of segments in this arrangement, as they all originate at $s \cdot P$. Then, the order of points by $s_t = \frac{1}{2}s + \frac{1}{2}s'$ is equivalent to the ordering of points by $s'$, and Statements (2)-(3) are also satisfied.

Next, suppose there are points in *P* with unequal heights with respect to $s$, so on Line 4, we let $h_1, h_2$ be the heights of points in *P* with the closest unequal heights in direction $s$. Additionally, we let $h_1', h_2'$ be the heights of points in *P* with the extreme heights in direction $s'$, as in Line 5. See Fig. 4. Consider the lines connecting $h_1$ to $h'_2$ and $h_2$ to $h_1'$; they intersect at some point *i* that is at least as close to the left as the leftmost non-zero intersection of all linear interpolations of point heights with respect to directions $s$ and $s'$; see Fig. 4. Let $t_*$ denote the first coordinate of *i* as on Line 6. Because $\frac{1}{2}t_* < t_*$, segments in the interval $(0, \frac{1}{2}t_*]$ of linear interpolations of point heights must not have any crossings, and so the ordering of points that have unique heights with respect to $s$ is maintained, i.e., $s_t$ satisfies Statement (1). Furthermore, if points have the same height with respect to direction $s$, in the linear interpolation of point heights, this corresponds to an intersection at $t=0$, so $s_t$ orders points equivalently to how $s'$ orders the points, satisfying Statements (2)-(3).

The runtime is dominated by the else statement on Line 3. To find $h_1, h_2, h_1',$ and $h_2'$, we sort the heights of points with respect to directions $s$ and $s'$ in $\Theta (|P |\log |P |)$ time. Finding the intersection $t_*$ of the resulting two segments takes constant time, and returning the direction $s_t = (1-\frac{1}{2}t_*) s+ \frac{1}{2}t_* s'$ takes $\Theta (d)$ time. Thus, the total runtime is $\Theta (|P |\log |P |+ d)$. $\square$

***Tilting to Pop***

Given a point set $P \subset \mathbb {R}^d$ in general position, two sets $W \subseteq V \subseteq P$, and a direction $s$ that is *P*-perpendicular to *V*, Algorithm 6 calculates a direction that is close to $s$ that “pops” the points of *W* above the points of $V\setminus W$.

**Algorithm 6:**
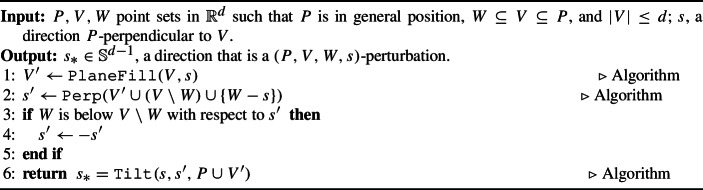
$$\texttt {TiltToPop}(P, V, W, s)$$

The algorithm begins by finding a set of points $V' \subset \mathbb {R}^d$ such that the affine space spanned by $V' \cup V$ is a $(d-1)$-dimensional subspace of $\mathbb {R}^d$ in $\Theta (d^3)$ time by Lemma 54. The additional points help us control which way to tilt *s*. In particular, the direction $s'$ (computed on Line 2 in $\Theta (d^3)$ by Lemma 53) is perpendicular to $\textrm{aff}(V' \cup (V\setminus W) \cup \{W-s\})$, and is the direction towards which we can tilt in order to “pop” *W* off of the plane orthogonal to $s$ at height $V \setminus W$. Because there are two choices for $s'$ in Line 2, the if statement in Lines 3 and 4 ensures that the direction is the one such that *W* is above $V\setminus W$, and this is completed in $\Theta (d)$ time. Finally, we return $s_*$ on Line 6 using Algorithm 5, taking $\Theta (|P |\log |P |+ d)$ by Lemma 29.

#### Lemma 30

(Tilting to Pop) Let *P* be a finite point set in $\mathbb {R}^d$ in general position. Let $W \subseteq V \subseteq P$ and $s\in \mathbb {S}^{d-1}$ such that $s$ is *P*-perpendicular to *V*. Then, Algorithm 6 calculates $\mathtt{{TiltToPop}}(P,V,W,s)$ in $\Theta (|P | \log |P | + d^3)$ time, and the output is a $(P, V, W, s)$-perturbation.

#### Proof

The runtime was justified in the paragraph above detailing the algorithm. Recall what it means for the returned direction to be a $(P, V, W, s)$-perturbation:

1. The points in *W* are above $V \setminus W$ with respect to direction returned.
2. For all $p \in P \setminus V$, *p* is strictly above (below) the height of $V \setminus W$ with respect to the direction returned if and only if it is strictly above (below, respectively) *V* with respect to $s$.
3. The direction returned is *P*-perpendicular to ${V\setminus W}$.

Before proving Statements (1)-(3), we establish three properties of $s'$ returned on Line 2. First, by Lemma 54, ${V'\cup V}$ consists of *d* points in general position, and *s* is normal to $\textrm{aff}(V'\cup V)$. Then, $\textrm{aff}(V' \cup (V \setminus W) \cup \{W -s\})$ is also $(d-1)$-dimensional, so $s'$ is automatically *P*-perpendicular to this space.

Next, we show that all points of *W* have the same height with respect to $s'$. If $W = \emptyset$, this is trivially true, so consider $w_1, w_2 \in W$. Because $(w_1-s), (w_2-s) \in \textrm{aff}(V' \cup (V \setminus W) \cup \{ W-s \})$, we have $s' \cdot (w_1 - s) = s' \cdot (w_2 - s)$. Then, by adding $s' \cdot s$ to both sides, we obtain $s' \cdot w_1 = s' \cdot w_2$, i.e., all points of *W* are at the same height with respect to $s'$.

Finally, we show $W \not \subset \textrm{aff}(V' \cup (V \setminus W) \cup \{ W-s \})$. Suppose not. Then, because both *W* and $W - s$ are in $\textrm{aff}(V' \cup (V \setminus W) \cup \{ W-s \})$, the vector $s$ is parallel to $\textrm{aff}(V' \cup (V \setminus W) \cup \{ W-s \})$. Furthermore, because both $\textrm{aff}(V' \cup (V \setminus W) \cup \{ W-s \})$ and $\textrm{aff}(V' \cup V)$ contain the same linearly independent set of $(d-1)$ points, namely $V' \cup V$, the planes are equal. However, this means *s* is parallel to $\textrm{aff}(V' \cup V)$, contradicting the property that $s$ is *P*-perpendicular to $\textrm{aff}(V' \cup V)$. Thus, we see that $s'$ orders all vertices of *W* on the same side of $V \setminus W$; on Line 4, we ensure that all vertices of *W* are specifically above $V \setminus W$ with respect to $s'$, therefore, by Lemma 29(2), all points of *W* are above the points of $V \setminus W$ with respect to the direction $s_*$ returned on Line 6 and we have shown $s_*$ satisfies Statement (1).

We must also show $s_*$ satisfies Statements (2) and (3) . Because both $s$ and $s'$ are perpendicular to $\textrm{aff}(V \setminus W)$, the output $\mathtt{{Tilt}}(s,s',P \cup V') = s_*$ is perpendicular to $\textrm{aff}(V\setminus W)$ by Lemma 29(3). To see that no other vertex of *P* is at the same height of vertices in $V \setminus W$ with respect to $s_*$, let $p \in P \setminus (V \setminus W)$. If $p \in P \setminus V$, because *p* is strictly above or below $V \setminus W$ with respect to direction $s$, then by Lemma 29(1), *p* is strictly above or below $V \setminus W$ with respect to $s_*$, showing Statement (2). If $p \in W$, then it is at the same height as $V \setminus W$ in direction $s$ and above $V \setminus W$ in direction $s'$, thus, by Lemma 29(2), *p* is above $V \setminus W$ in direction $s_*$ and we have shown that $s_*$ is *P*-perpendicular to $V \setminus W$, so Statement (3) of the current lemma is satisfied. $\square$

### Building an Explicit Set

Next, we use the algorithms of Section 5.1 to construct vertex- and simplex-isolating directions for a given simplicial complex.

#### Directions for Vertices

Constructing a set of directions that faithfully represents a vertex set has been explored in previous work. By [3, Lemma 7], it suffices to construct a set of *d* linearly independent directions, plus one additional direction so that there are exactly $n_0$ intersections of size $d+1$ among all associated filtration hyperplanes. However, the construction of this final direction given in [3, Lemma 8] requires stricter general position assumptions to construct the set, namely, that no two vertices share any $e_i$-coordinate for $1 \le i \le d$. Here, we provide an algorithm to produce such a $(d+1)$st direction when our pointset satisfies only the mild general position assumptions described in the “General Position” paragraph of Section 2.1.

**Algorithm 7:**
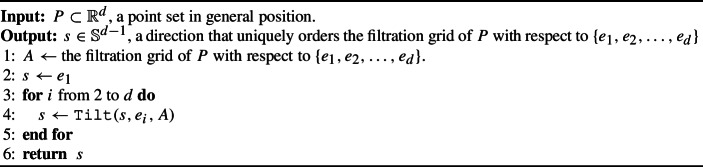
$$\mathtt{{PointIso}}(P)$$

The next lemma proves correctness of Algorithm 7.

##### Lemma 31

(Correctness of Algorithm 7) Let *P* be a finite point set in $\mathbb {R}^d$ in general position. Then, $\mathtt{{PointIso}}(P)$ returns a direction that uniquely orders the filtration grid of *P* with respect to $\{e_1, e_2, \ldots , e_d\}$ and runs in $\Theta (d^2 \vert P \vert ^d \log \vert P \vert )$ time.

##### Proof

On Line 4, we call Algorithm 5, which by Lemma 29 takes $\Theta (\vert A\vert \log \vert A\vert + d)$ time. This is called $d-1$ times in the loop of Lines 3–5; because $\vert A\vert = \Theta (\vert P\vert ^d)$, Algorithm 7 takes $\Theta (d(\vert P \vert ^d \log \vert P \vert ^d + d)) = \Theta (d^2 \vert P \vert ^d \log \vert P \vert )$ total time.

Next, we show Algorithm 7 is correct. Let $\pi _i$ be the standard projection map onto the $(e_1, e_2, \ldots e_i$)-plane. As on Line 1, let *A* be the filtration grid of *P* with respect to $\{e_1, e_2, \ldots , e_d\}$ and note that *A* is a grid of at most $\vert P \vert ^d$ points. Let *j* be the number of times the loop has been completed. We use the loop invariant that, in Lines 3–5, $s$ totally orders the unique points of the image $\pi _{j+1}(A)$. We first show this is true before entering the loop. We initialize $s= e_1$ on Line 2. Thus, because $e_1$ totally orders the points of $\pi _1(A)$, the loop invariant is satisfied.

Let $s^i$ denote the *i*th value of $s$ in Algorithm 7 (so $s^1$ is the initial direction defined in Line 2, $s^2$ is the direction updated by tilting towards $e_2$ the first time we encounter Line 3, etc. Note that this means $j=i-1$.)

Suppose that the loop invariant is true going into the for loop of Lines 3–5. Recall by Lemma 29 that $\mathtt{{Tilt}}(s^{i-1},e_{i},A)$ produces a direction $s^{i}$ so that, for all $a_1, a_2 \in A$,

1. If $a_1$ is strictly above (below) $a_2$ with respect to direction $s^{i-1}$, then $a_1$ is strictly above (below, respectively) $a_2$ with respect to direction $s^{i}$.
2. If $a_1$ and $a_2$ are at the same height with respect to direction $s^{i-1}$ and $a_1$ is strictly above (below) $a_2$ with respect to direction $e_{i}$, then $a_1$ is strictly above (respectively, below) $a_2$ with respect to direction $s^{i}$.
3. If $a_1$ is at the same height as $a_2$ with respect to both directions $s^{i-1}$ and $e_{i}$, then $a_1$ and $a_2$ are at the same height with respect to direction $s^{i}$.

Because $s^{i-1}$ provided a total order of $\pi _{i-1}(A)$ by assumption and given the statements above, we conclude that $s^i$ totally orders $\pi _i(A)$. Suppose that after the loop terminates, $s^d = \mathtt{{PointIso}}(P)$, totally orders the points of $\pi _d(A)$. Then, because $\pi _d(A) = A$, the final direction totally orders the points of *A*. Finally, by the runtime analysis, the loop terminates and thus, Algorithm 7 is correct. $\square$

Using the previous lemma, we are now able to construct a set of vertex-isolating directions. This is a generalization of [3, Lemma 7 and Theorem 9] and we give a brief restatement of the main idea of the proof.

##### Lemma 32

(Construction of Vertex-Isolating Directions) Let $K \subset \mathbb {R}^d$ be a GP-immersed simplicial complex. Then, the basis directions $e_1, e_2, \ldots , e_d$, along with the direction $s= \mathtt{{PointIso}}(K_0)$, are vertex-isolating.

##### Proof

Let *A* denote the filtration grid of $K_0$ with respect to $\{e_1, e_2, \ldots , e_d\}$. Recall that $\mathbb {H}(s, K_0)$ is the set of filtration hyperplanes of $K_0$ with respect to *s* (Definition 7). Because $s$ orders the points of *A* uniquely by Lemma 31, we know by [3, Lemma 7] that the vertices $K_0$ are in one-to-one correspondence with the points $\mathbb {H}(s, K_0) \cap A$. Briefly, this is because $\mathbb {H}(s, K_0)$ has a unique hyperplane passing through each point of $K_0$. Then, because each point of $K_0$ lies on some point of *A* , and because no hyperplane of $\mathbb {H}(s, K_0)$ passes more than one point of *A*, we have $\mathbb {H}(s, K_0) \cap A = K_0$. $\square$

#### Directions for Higher-Dimensional Simplices

Next, we show how auxiliary constructions of Section 5.1 can be used to construct sets of directions that are simplex-isolating. If a simplex $\sigma$ is less than $(d-1)$-dimensional, the direction returned by $\mathtt{{Perp}}(\sigma )$ is not guaranteed to be $K_0$-perpendicular to $\sigma$. Thus, to ensure we have a direction that places $\sigma$ at a unique height, we “pop” off any extra vertices using $\mathtt{{TiltToPop}}$, returning a tilted direction that is guaranteed to be $K_0$-perpendicular to $\sigma$.

##### Lemma 33

(Construction of $K_0$-Perpendicular Directions) Let $K$ be a simplicial complex GP-immersed in $\mathbb {R}^d$. Let $\sigma$ be a simplex of $K$. Furthermore, let $s= \mathtt{{Perp}}(\sigma )$, let *V* denote the set of all vertices with the same height as $\sigma$ in direction $s$, and let $W = V \setminus \sigma$. Then $\mathtt{{TiltToPop}}(K_0,V,W,s)$ is $K_0$-perpendicular to $\sigma$. Furthermore, this direction can be computed in $O((n_0+d) \log (n_0+d) + d^3)$ time.

##### Proof

By construction, *V* is the set of all vertices with height $s \cdot \sigma$, meaning that *s* is indeed $K_0$-perpendicular to *V*. The other inputs to $\mathtt{{TiltToPop}}$ are also valid by construction. Then, by Lemma 30, the direction returned by $\mathtt{{TiltToPop}}(K_0,V,W,s)$ is a $(K_0, V, W, s)$-perturbation. In particular, by Statement (1) of Definition 10, this means the direction is $K_0$-perpendicular to $V \setminus W = \sigma$, as desired.

We compute $s= \mathtt{{Perp}}(\sigma )$ in $\Theta (d^3)$ time by Lemma 53. We find *V* and *W* in $O(n_0)$ time. Finally, we compute $\mathtt{{TiltToPop}}(K_0,V,W,s)$. The algorithm $\mathtt{{TiltToPop}}$ has runtime $\Theta ((n_0+|W |) \log (n_0+|W |) + d^3)$ by Lemma 30. Because *W* has *O*(*d*) vertices, all these operations in total take $O((n_0+d) \log (n_0+d) + d^3)$ time. $\square$

Putting the pieces together, we use the algorithms of this section and Section 5.1 to construct a set of directions.

##### Construction 34

(Constructing a Faithful Set) Let $K$ be a simplicial complex GP-immersed in $\mathbb {R}^d$ such that $\dim (K) < d$, and let *S* be the set of directions constructed iteratively as follows:

1. Initially, let *S* be the standard basis vectors ($e_1$, $e_2$, ..., $e_d$), plus the additional direction $\mathtt{{PointIso}}(K_0)$.
2. For every maximal $\sigma \in K$,
  - Let $s= \mathtt{{Perp}}(\sigma )$, let *V* be the set of all vertices with the same height as $\sigma$ in direction $s$, and let $W = V \setminus \sigma$. Then, add the direction $s_{\sigma }:= \mathtt{{TiltToPop}}(K_0,V,W,s)$ to *S*.
  - For each $\tau \in K$ such that $\tau \prec \sigma$, add the direction $\mathtt{{TiltToPop}}(K_0,\sigma ,\tau ,s_{\sigma })$ to *S*.

Finally, we show that Construction 34 forms a faithful discretization of the VPHT, the VBT, and the VECT, and analyze the size and time complexity.

##### Theorem 35

(Explicit Faithful Discretization) Let $K$ be a simplicial complex GP-immersed in $\mathbb {R}^d$ such that $\dim (K) < d$, and let *S* be the set of directions from Construction 34. Let $\widetilde{\mathcal {A}}\in \{\widetilde{\mathcal {D}}, \widetilde{\beta }, \widetilde{\chi }\}$. Then, $\widetilde{\mathcal {A}}[K,S]$ is a faithful discretization of $\widetilde{\mathcal {A}}[K,\mathbb {S}^{d-1}]$ of size $O(n2^{{\dim (K)}}+d)$, and *S* can be computed in $O(\log (n_0 + d) (d^2 n_0^d + nd + nn_0 2^{\dim (K)}) + n2^{\dim (K)}d^3)$ time.

##### Proof

By Lemma 31, the directions added to *S* in Step 1 are vertex-isolating (Definition 11). By Lemma 33, the directions added to *S* in Step 2a are $K_0$-perpendicular to every maximal simplex, satisfying the first condition of being simplex-isolating (Definition 12(1)). By Lemma 30, the directions added to *S* in Step 2b are $(P, V, W, s)$-perturbations, satisfying the second condition of being simplex-isolating (Definition 12(2)). Thus, the directions of Step 2 are simplex-isolating. Because (*K*, *S*) is both vertex- and simplex-isolating, by Theorem 14, $\widetilde{\mathcal {A}}[K,S]$ is a faithful discretization of $\widetilde{\mathcal {A}}[K,\mathbb {S}^{d-1}]$.

Now, we analyze size and time bounds. By Lemma 31, the $\Theta (d)$ directions added to *S* in Step 1 can be computed in time $\Theta (d^2 n_0^d \log n_0)$.

Next, we give bounds for Step 2a. By Lemma 33, for each maximal $\sigma \in K$, the direction $s_{\sigma }$ in Step 2a can be computed in time $O((n_0+d) \log (n_0+d) + d^3)$. Because the total number of maximal simplices is *O*(*n*), the total time computing directions in Step 2a is $O\left( n((n_0+d) \log (n_0+d) + d^3)\right)$.

Given $s_{\sigma }$ and $\tau \prec \sigma$, by Lemma 30, a single direction in Step 2b can be computed in time $\Theta (n_0 \log n_0 + d^3)$. Because, for every maximal *i*-simplex of $K$, we compute one direction for each of its proper faces, each *i*-simplex adds a total of $2^{i+1}-2$ directions in Step 2b. Then, the number of directions in Step 2b is $\Theta (n2^{{\dim (K)}})$. Hence, the total time to compute directions in Step 2b is $O(n2^{\dim (K)}(n_0 \log n_0 + d^3))$.

Thus, the set *S* has $O(n2^{{\dim (K)}}+d)$ directions and can be computed in time $O(\log (n_0 + d) (d^2 n_0^d + nd + nn_0 2^{\dim (K)}) + n2^{\dim (K)}d^3)$. $\square$

## Stability Results for Faithful Discretizations

**Fig. 5:**
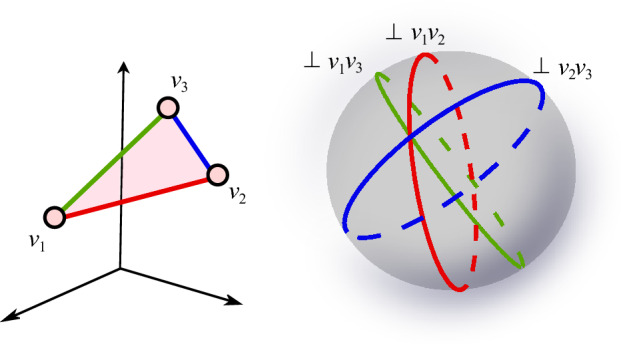
Three vertices in $\mathbb {R}^3$ (left) divide $\mathbb {S}^2$ into 14 strata. Each stratum is a region containing all directions that define the same partial order on vertices. Note that the set of directions perpendicular to the line defined by two vertices forms a great circle; the two directions perpendicular to $[v_1,v_2,v_3]$ correspond to the two three-way intersections of these great circles

In this section, we investigate the stability of our discretization under perturbations of directions and vertices. Central to these results is the stratification of the sphere of directions induced by directional transforms, as has been noted in related work, e.g., [1, 11, 16, 19, 26].^3^ The stratification is defined by dividing the sphere of directions into regions (strata) where all directions within the same stratum induce the same partial order on vertices (ordered by their height with respect to that direction); see Fig. 5.

We begin by defining a strata-preserving map, which allows for a clean way to describe important relationships between sets of directions.

### Definition 36

(Strata-Preserving Map) Given $S \subseteq \mathbb {S}^{d-1}$ and a simplicial complex $K$ GP-immersed in $\mathbb {R}^d$, the map $f: \mathbb {S}^{d-1} \rightarrow \mathbb {S}^{d-1}$ is *strata-preserving with respect to* *K* *and* *S* if, for every $s\in S$, the partial orderings of vertices induced by their heights with respect to directions $s$ and $f(s)$ are the same.

Next, we give conditions under which a set of directions may be perturbed and still correspond to a faithful discretization.

### Theorem 37

(Directional Perturbations) Let $\widetilde{\mathcal {A}}\in \{\widetilde{\mathcal {D}}, \widetilde{\beta }, \widetilde{\chi }\}$, let $K$ be a simplicial complex GP-immersed in $\mathbb {R}^d$, and let $S \subseteq \mathbb {S}^{d-1}$ be such that (*K*, *S*) is vertex- and simplex-isolating. Let $f :\mathbb {S}^{d-1} \rightarrow \mathbb {S}^{d-1}$ be strata-preserving with respect to *K* and *S*. If (*K*, *f*(*S*)) is vertex-isolating, then $\widetilde{\mathcal {A}}[K,f(S)]$ is a faithful discretization of $\widetilde{\mathcal {A}}[K,\mathbb {S}^{d-1}]$.

### Proof

By hypothesis, the set (*K*, *f*(*S*)) is vertex-isolating. Let $\sigma$ be a maximal simplex. Because (*K*, *S*) is simplex-isolating, *S* contains a direction $s_{\sigma }$ that is $K_0$-perpendicular to $\sigma$. Because *f* is strata-preserving, it preserves vertex order, so $f(s_{\sigma })$ is also $K_0$-perpendicular to $\sigma$. Moreover, for $\tau \prec \sigma$ and for a $(K_0,\sigma ,\sigma \setminus \tau ,s_{\sigma })$-perturbation $s' \in S$, the direction $f(s')$ is a $(K_0,\sigma ,\sigma \setminus \tau ,f(s_{\sigma }))$-perturbation. Hence, (*K*, *f*(*S*)) is $\sigma$-isolating. Because $\sigma$ was arbitrarily chosen, we know that (*K*, *f*(*S*)) is simplex-isolating. Because $(K,f(S))$ is vertex- and simplex-isolating, by Theorem 14, $\widetilde{\mathcal {A}}[K,f(S)]$ is a faithful discretization of $\widetilde{\mathcal {A}}[K,\mathbb {S}^{d-1}]$. $\square$

Given a simplicial complex *K* immersed in $\mathbb {R}^d$ and $\epsilon >0$, we call *L* an $\epsilon$-*perturbation of* *K* if there exists a simplicial homeomorphism $g :K \rightarrow L$ such that, for all $v \in K_0$, we have $|v-g(v)|\le \epsilon$. Vertex heights change continuously when moving a vertex continuously; the next result makes use of this fact.

### Theorem 38

(Perturbations of Immersion) Let $\widetilde{\mathcal {A}}\in \{\widetilde{\mathcal {D}}, \widetilde{\beta }, \widetilde{\chi }\}$, let $K$ be a simplicial complex GP-immersed in $\mathbb {R}^d$, and let *S* be as in Construction 34. Let *L* be an $\epsilon$-perturbation of *K* such that, with respect to every $s\in S$, the order of vertex heights in $L_0$ with is the same order of vertex heights in $K_0$. Then, for $\epsilon$ sufficiently small, $\widetilde{\mathcal {A}}[L,S]$ is a faithful discretization of $\widetilde{\mathcal {A}}[L,\mathbb {S}^{d-1}]$.

### Proof

Because *L* is obtained from *K* by perturbing the vertices, we have a homeomorphism and simplicial map $g :K \rightarrow L$ such that, for a vertex $v \in K_0$, *g*(*v*) is the perturbation of *v*. Let $\sigma \in K$ be a maximal simplex. Because (*K*, *S*) is $\sigma$-isolating, there is a direction $s_{\sigma } \in S$ that is $K_0$-perpendicular to $\sigma$. Because *K* and *L* have the same vertex order with respect to direction $s_{\sigma }$, we know $s_{\sigma }$ is $L_0$-perpendicular to $g(\sigma )$. Moreover, for $\tau \prec \sigma$, if $s'$ is a $(K_0,\sigma ,\sigma \setminus \tau ,s_{\sigma })$-perturbation, then $s'$ is also a $(g(K_0),g(\sigma ),g(\sigma \setminus \tau ),s_{\sigma })$-perturbation. Hence, $(g(K),S)=(L,S)$ is $g(\sigma )$-isolating. Because *g* is a bijection, (*L*, *S*) is simplex-isolating.

**Fig. 6:**
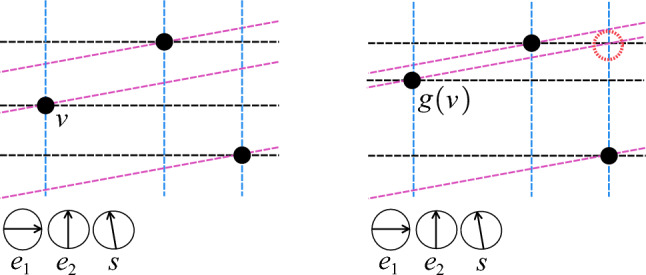
The direction *s* uniquely orders the filtration grid of the leftmost vertex set with respect to $\{e_1, e_2\}$, meaning $\{e_1, e_2, s\}$ is vertex-isolating. On the right, we see that even an $\epsilon$-perturbation that preserves vertex orders in each direction does not guarantee that the set remains vertex-isolating; the new position of *g*(*v*) causes an extra intersection (in the red dotted circle), so *s* no longer uniquely orders the filtration grid and $\{e_1, e_2, s\}$ is no longer vertex-isolating. However, for small enough $\epsilon$, such extra intersections are avoided

Next, suppose that $s_1, s_2, \ldots , s_{d+1} \in S$ are vertex-isolating directions for *K*; in particular, $s_{d+1}$ orders $K_0$ uniquely. Consider a linear interpolation between $K_0$ and $L_0$ induced by *g*, and thus, a continuous interpolation between the arrangement of filtration hyperplanes of *K* and *L* with respect to the directions $\{s_1, s_2, \ldots , s_{d+1}\}$. If $\epsilon$ is small enough, no topological changes occur in the interpolation of these arrangements, i.e., $s_{d+1}$ still orders the filtration grid of *L* with respect to the directions $\{s_1, s_2, \ldots , s_d\}$ uniquely, so (*L*, *S*) is vertex-isolating. See Fig. 6 for a potential issue that arises if we do not choose $\epsilon$ small enough.

Thus, because (*L*, *S*) is vertex- and simplex-isolating, by Theorem 14, $\widetilde{\mathcal {A}}[L,S]$ faithfully discretizes $\widetilde{\mathcal {A}}[L,\mathbb {S}^{d-1}]$ as desired. $\square$

## Discussion

In this work, we provide sufficient conditions for a faithful discretization of the verbose persistent homology transform. Because only the presence and dimension of filtration events are used (and not birth/death pairing information), the techniques presented in the paper immediately apply to the verbose Betti function transform. In fact, by the same reasoning, they apply to *any* verbose directional transform that contains information about the dimension and height of simplices. Additionally, we show that, under mild adaptations, the methods of this paper also yield a faithful discretization of the verbose Euler Characteristic function transform. This is particularly important because, in practice, VECFs are often preferred to VPDs due to the existence of faster algorithms for computing the functions.

For a simplicial complex GP-immersed in $\mathbb {R}^d$ with $n_0$ vertices, *n* simplices, and dimension ${\dim (K)}$, we compute a faithful discretization of size $O(n2^{{\dim (K)}}+d)$ in $O(\log (n_0 + d) (d^2 n_0^d + nd + nn_0 2^{\dim (K)}) + n2^{\dim (K)}d^3)$ time. We go on to show that the discretization is stable with respect to multiple types of perturbations. In particular, the size of our discretization remains unchanged by such perturbations and is independent of any restrictions on the simplicial complex beyond standard general position assumptions.

The properties and explicit constructions of faithful discretizations computed in this paper are an important step for use in both applications and theory. While our use of verbose descriptors results in a generally much smaller discretization than the best-known bounds for discretizations that use concise descriptors, the sets discussed in this paper are often still much larger than is strictly necessary to form a faithful discretization. In ongoing work, we hope to better understand computing faithful discretizations with minimal cardinality.

## Figures and Tables

**Algorithm 8:**
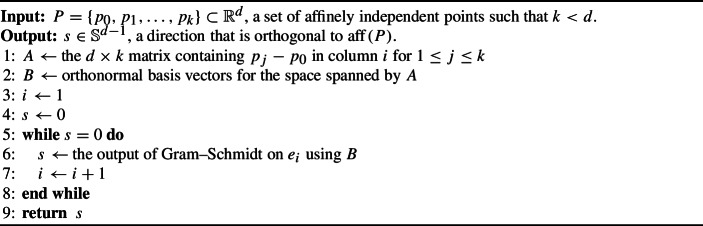
$$\mathtt{{Perp}}(P)$$

**Algorithm 9:**
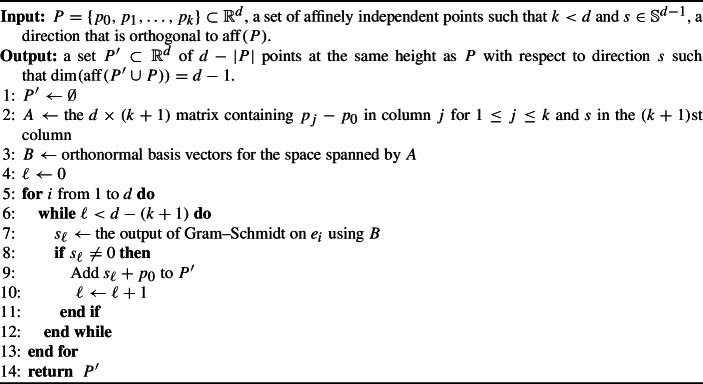
$$\mathtt{{PlaneFill}}(P,s)$$

**Fig. 7:**
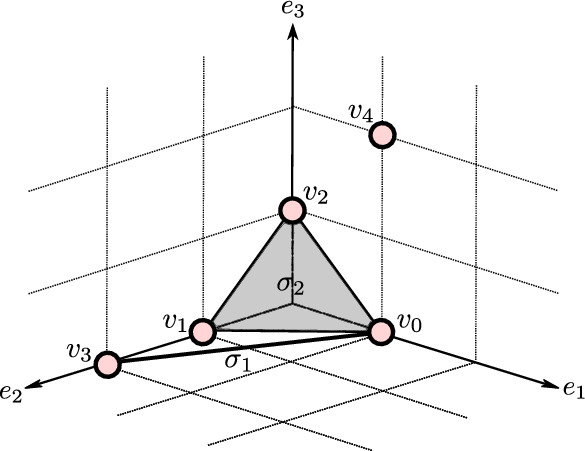
The simplicial complex *K* used as an example in this section. We first find vertex-isolating directions: the basis vectors, along with a fourth direction that orders the filtration grid of $K_0$ with respect to the basis vectors uniquely. Next, for $\sigma _1$ and $\sigma _2$, we find simplex-isolating directions; directions that are $K_0$-perpendicular to $\sigma _1$ and $\sigma _2$, as well as directions that tilt these perpendicular directions so that proper faces of $\sigma _1$ (and $\sigma _2$) are “popped” above $\sigma _1$ (respectively, $\sigma _2$)

## Concise Versus Verbose

In this appendix, we briefly make contact with the work of Curry, Mukherjee, and Turner [11], which explores the existence of a faithful discretization of the *concise* PHT and ECT. In particular, in Theorem 7.14, we see a bound on the size of a faithful discretization of concise Euler characteristic functions or concise persistence diagrams when, where assumptions about the underlying simplicial complex must be made.

Roughly speaking, the bound of [11] is stated for simplicial complexes that are known to satisfy two constraints ensuring *K* is not too “flat,” or that no collection of points are too close to coplanar. First, we give a notion of criticality, which is restated from [11, Def. 7.2].

### Definition 39

(Observable) Let *K* be a simplicial complex embedded in $\mathbb {R}^d$, and consider a direction $s \in \mathbb {S}^{d-1}$. We say that a vertex $v \in K_0$ is *observable by* *s* if the concise Euler characteristic function changes at $s \cdot v$.

Following [11, Def. 7.5]), given a dimension *d* and parameters $\delta \in \mathbb {R}_+$ and $k \in \mathbb {Z}_+$, we define a family of simplicial complexes.

### Definition 40

(Well-Behaved Families) Let $\mathcal {K}(d,\delta ,k)$ denote the set of simplicial complexes *K* satisfying:

1. *K* is embedded in $\mathbb {R}^d$.
2. For each $v \in K_0$, there exists a ball $B \subset \mathbb {S}^{d-1}$ of radius $\delta$ such that for all $s \in B$, *v* is observable by *s*.
3. For each $s \in \mathbb {S}^{d-1}$, consider the ball $B_s \subset \mathbb {S}^{d-1}$ of radius $\delta$ centered at *s*. Then, the number of vertices observable by a direction $s' \in B_s$ is never more than *k*.

Then, we have the following bound on the size of a concise discretization.

### Theorem 41

[11, Thm. 7.14] Given a dimension *d* and two parameters $\delta \in \mathbb {R}_+$ and $k \in \mathbb {Z}_{+}$, for any shape in $\mathcal {K}(d, \delta , k)$, there exists a set of

A1 $$\begin{aligned} ((d-1)k +1) \bigg (1 + \frac{3}{\delta }\bigg )^d + O\bigg (\frac{d^{d+1}k^{2d}}{\delta ^{2d(d-1)}} \bigg ) \end{aligned}$$

directions whose discretization of the ECT or PHT faithfully represents the shape, when restricted to shapes in $\mathcal {K}(d, \delta , k)$.

Although the above theorem requires knowing or assuming $\delta$ and *k* a priori, and thus takes a rather different approach than our current results, we still see an interesting consequence of using concise versus verbose descriptors through comparison. The following example highlights perhaps the most important difference of our results: sensitivity versus insensitivity to the “flatness” of *K*.

### Example 42

*(Sensitivity/Insensitivity to Flatness)* Consider the simplicial complex *K* consisting of two adjacent edges in $\mathbb {R}^2$, $[v_0,v_1]$, and $[v_1, v_2]$ (this is a simplification of [11, Ex. 7.4]). Denote the angle between the edges by $\theta$. Then, $v_1$ is only observable by directions that order it first or last. Such directions define a ball *B* with radius $\delta = \pi - \theta$. Because $v_0$ and $v_2$ are observable in balls with larger radius, we see that we must use $\delta \le \pi - \theta$ as our parameter in Statement (2) of Definition 40.

Because the Euler characteristic can only change at a vertex height, and all three vertices are indeed observable for some directions, we must use $k \ge 3$ in Statement (3). Then, the bound given in [11] becomes

A2 $$\begin{aligned} \begin{aligned}&((2-1)k +1) \bigg (1 + \frac{3}{(\pi - \theta )}\bigg )^2 + O\bigg (\frac{2^{3}k^{4}}{(\pi - \theta ) ^{4(1)}} \bigg )\\&\quad = (k+1) \bigg (1 + \frac{3}{(\pi -\theta )}\bigg )^2 + O\bigg (\frac{k^4}{(\pi -\theta ) ^{4}}\bigg ). \end{aligned} \end{aligned}$$

By moving $v_0$, $v_1$, and $v_2$ closer to colinear, $\theta$ approaches $\pi$. Notice this does not change the bound $k \ge 3$, because, as long as the vertices are not quite colinear, there is always some direction that sees three changes to the Euler characteristic. Thus, simply by adjusting vertex locations slightly, we can construct examples where the value of Equation (A2) is as large as we would like.

On the other hand, by Theorem 14, we only require $O(n2^{{\dim (K)}} + d)$ directions when using verbose descriptors. Specifically, this is $d+1= 3$ vertex-isolating directions and six simplex-isolating directions (for each edge, one perpendicular direction and two perturbed directions). Thus, our total number of required directions is independent of the angle at $v_1$ and remains constant at nine.

Note that the example above is related to the stable behavior for verbose descriptors described in Theorem 38. While the bound of [11] is not shown to be tight, sensitivity to the structure of *K* when using concise descriptors is unavoidable. Despite their trivial lifespans, the “on-diagonal” points of a verbose persistence diagram (or, the analogue of such points in a verbose Euler characteristic function) have a significant impact on the behavior of faithful sets. A thorough exploration of the differences between concise and verbose descriptors is outside of the scope of this paper, so we refer the reader to [13, 15, 29, 42]. In particular, sensitivity of concise descriptors to the structure of *K* is highlighted in [15, Section 6], which shows, if we do not restrict $\delta$ or *k*, faithful sets of concise descriptors require multiple directions perpendicular to each maximal simplex.

## Verbose Descriptors are Well-Defined

We begin by showing VPDs are well-defined. Existence of VPDs compatible with a PD follows from the fact that any partial order (namely, the partial order induced by a general filtration) admits a compatible linear extension (corresponding to the total order of a compatible index filtration). We therefore focus on showing uniqueness of the corresponding VPD. This boils down to proving that Definition 1 is independent of the choice of the index filtration. To prove this claim, we will make use of the following definition, which organizes collections of *f*-compatible filter functions.

### Definition 43

*(Compatible Transposition Sequence)* Let $K$ be a simplicial complex and let $f :K \rightarrow \mathbb {R}$ a filter function. Let $f_1, f_2, \ldots , f_m$ be *f*-compatible index filtrations, where each adjacent pair $f_j$ and $f_{j+1}$ differ only by a single transposition. That is, for some $i \in \mathbb {N}$, we have the equalities $f_j^{-1}(i) = f_{j+1}^{-1}(i+1)$ and $f_j^{-1}(i+1) = f_{j+1}^{-1}(i)$, but for every other $i' \not \in \{i,i+1\}$, we have $f_j^{-1}(i') = f_{j}^{-1}(i')$. This sequence of such index filtrations is called an *f*-*compatible transposition sequence*.

We begin by considering transposition sequences with just two elements.

### Lemma 44

(Commutative Diagrams) Let $K$ be a simplicial complex and $f :K \rightarrow \mathbb {R}$ be a filter function. Let $\{f_1, f_2\}$ be an *f*-compatible transposition sequence, where the transposition between $f_1$ and $f_2$ occurs at indices *i* and $i+1$. Let $\{K_j^{(1)}\}_{j \in \mathbb {N}}$ and $\{K_j^{(2)}\}_{j \in \mathbb {N}}$ denote the corresponding filtrations. Then, the following diagram of inclusions commutes:

### Proof

The proof follows from the fact that all maps are inclusion maps.

Applying homology to Equation (B3), we obtain the following corollary.

### Corollary 45

(Commutative Diagrams on Homology) The following diagram commutes:

Suppose that $f_1$ and $f_2$ are *f*-compatible index filtrations that differ by a single transposition. We show that it does not matter if we compute *f* using $f_1$ or $f_2$. Following [9, p. 122], there are four cases to consider, namely, whether births or deaths occur at index *i* and $i+1$. We first consider the case that *i* and $i+1$ both correspond to birth events; the other cases are similar.

### Lemma 46

(Transpose Births) Let $K$ be a simplicial complex and $f :K \rightarrow \mathbb {R}$ be a filter function. Let $f_1$ and $f_2$ be two *f*-compatible index filters that differ by exactly one transposition at index *i* and $i+1$. If both *i* and $i+1$ are birth coordinates in $\mathcal {D}_{f_1}$, then

$$\left\{ \left( f\left( f_1^{-1}(i)\right) , f\left( f_1^{-1}(j)\right) \right) \right\} _{(i,j) \in \mathcal {D}_{f_t}} = \left\{ \big (f(f_2^{-1}(i)), f(f_2^{-1}(j)) \big ) \right\} _{(i,j) \in \mathcal {D}_{f_1}},$$

with $f(\emptyset ):=\infty$ and where $=$ denotes equality as a multi-set.

### Proof

For $j \in \{1,2,\ldots , n\}$, let $\sigma _j:= f_1^{-1}(j)$; in other words, $\sigma _1,\sigma _2, \ldots , \sigma _n$ is the ordering of the simplices such that $f_1(\sigma _j)=j$. Because the transposition is at indices *i* and $i+1$, we have $f_1(\sigma _i)=f_{2}(\sigma _{i+1})=i$ and $f_{2}(\sigma _{i+1})=f_1(\sigma _{i+1})=i+1$. For all other $\sigma ' \in K$, we have $f_1(\sigma ')=f_{2}(\sigma ')$.

Because *i* is a birth coordinate in $\mathcal {D}_{f_1}$, and because only a single simplex is added at index *i*, we know $\text {rk}(H(K_{i}^{(1)})) = \text {rk}(H(K_{i-1}^{(1)})+1$ (where $\text {rk}(H(-))$ denotes the sum of homology ranks over all dimensions). Likewise, $\text {rk}(H(K_{i+1}^{(1)}) = \text {rk}(H(K_{i}^{(1)})+1$. See, e.g., [12, Chapter V.4, pp. 120–121]. Thus, for all $k \in \mathbb {N}$, and using Corollary 45, we have

B5 $$\begin{aligned} \text {rk}\,(H_k(K_{i+1}^{(2)}) =\text {rk}\,(H_k(K_{i+1}^{(1)})) = \text {rk}\,(H(K_{i-1}^{(1)})) +2 = \text {rk}\,(H_k(K_{i-1}^{(2)}))+2. \end{aligned}$$

Then, because the rank of homology groups only increases and because $K_{i-1}^{(2)}$ and $K_{i+1}^{(2)}$ differ by two simplices, we know that both *i* and $i+1$ are also birth coordinates in $\mathcal {D}_{f_2}$.

For $k \in \mathbb {N}$ and $s,t \in \overline{\mathbb {R}}$, let $\beta _{k,s,t}^{(1)} = \beta _{k,s,t}(f_{1})$ and $\beta _{k,s,t}^{(2)} = \beta _{k,s,t}(f_{2})$. By using $f_1$ for the filter in Definition 1 (and rephrasing Equation (1) specifically for index filtrations) the multiplicity of $(s',t')$ in $\widetilde{\mathcal {D}}_{f, k}$ is:

B6 $$\begin{aligned} \mu _k^{(1)}(s',t')&= \sum _{\begin{array}{c} (s,t) \in \overline{\mathbb {R}}^2 \\ s < t \\ f(f_1^{-1}(s))=s'\\ f(f_1^{-1}(t))=t' \end{array}} \mu _k^f(s,t) \end{aligned}$$

B7 $$\begin{aligned}&= \sum _{\begin{array}{c} (s,t) \in \overline{\mathbb {R}}^2 \\ s< t\\ f(f_1^{-1}(s))=s'\\ f(f_1^{-1}(t))=t' \end{array}} \left( \beta _{k,s,t-1}^{(1)} - \beta _{k,s,t}^{(1)} - \beta _{k,s-1,t-1}^{(1)} + \beta _{k,s-1,t}^{(1)} \right) . \end{aligned}$$

Similarly, let $\mu _k^{(2)} :\overline{\mathbb {R}}^2 \rightarrow \mathbb {Z}$ map $(s',t')$ to the multiplicity of $(s',t')$ in $\widetilde{\mathcal {D}}_{f, k}$, as defined using $f_2$ as the filter in Definition 1. Now, we must show that $\mu _k^{(1)}=\mu _k^{(2)}$ for all *k*.

We investigate the values of $\beta _{k,s,t}^{(1)}$ for different values of *s* and *t*. Because we are working with index filters, $\beta _{k,s,t}^{(1)} = \beta _{k,\lfloor s \rfloor ,\lfloor t \rfloor }^{(1)}$ and it is sufficient to only consider integer values of *s*, *t*. First, suppose $s \ne i$. Then, we also have $t \ne i$. Because Diagram B4 commutes, we know:

$$\begin{aligned} \beta _{k,s,t}^{(1)}&= \text {rk}\, \left( H_k( K_s^{(1)}) \rightarrow H_k( K_t^{(1)}) \right) \\&= \text {rk}\, \left( H_k( K_s^{(2)}) \rightarrow H_k( K_t^{(2)}) \right) \\&= \beta _{k,s,t}^{(2)}. \end{aligned}$$

Next, consider the case $s=i$. Because $s=i$ is a birth parameter, there exists $d_i \in \overline{\mathbb {R}}$ such that $(i,d_i) \in \mathcal {D}_{f_1}$. Similarly, let $d_{i+1} \in \overline{\mathbb {R}}$ such that $(i+1,d_{i+1}) \in \mathcal {D}_{f_1}$. Either let $t \ge \max \{d_i, d_{i+1}\}$, or $t < \min (d_i,d_{i+1})$, or suppose *k* does not equal the dimension of death at $\max \{d_i, d_{i+1}\}$. Then, loosely speaking, $K_i^{(1)} \hookrightarrow K_t^{(1)}$ and $K_i^{(2)} \hookrightarrow K_t^{(2)}$ either do not include the deaths at $d_i$ and $d_{i+1}$, or they include both deaths. Either way, we have,

$$\begin{aligned} \beta _{k,i,t}^{(1)}&= \text {rk}\, \left( H_k( K_i^{(1)}) \rightarrow H_k( K_t^{(1)}) \right)  &   \text {by definition} \\&= \text {rk}\, \left( H_k( K_i^{(2)}) \rightarrow H_k( K_t^{(2)}) \right) \\&= \beta _{k,i,t}^{(2)}. \end{aligned}$$

Now, consider the case that *k* equals the dimension of death at $\max \{d_i, d_{i+1}\}$, and a value $t \in \mathbb {N}$ such that $t \in [\min \{d_i,d_{i+1}\}, \max \{d_i, d_{i+1}\})$. First, suppose $d_i < d_{i+1}$, so that, loosely speaking, $K_i^{(1)} \hookrightarrow K_t^{(1)}$ includes the death at $d_i$ (but not at $d_{i+1}$), but $K_i^{(2)} \hookrightarrow K_t^{(2)}$ does not include the death at $d_{i}$ (nor at $d_{i+1}$). Then,

$$\begin{aligned} \beta _{k,i,t}^{(1)}&= \text {rk}\, \left( H_k( K_i^{(1)}) \rightarrow H_k( K_t^{(1)}) \right) \\&= \text {rk}\, \left( H_k( K_i^{(2)}) \rightarrow H_k( K_t^{(2)}) \right) + 1 \\&= \beta _{k,i,t}^{(2)} + 1 \end{aligned}$$

If *k* equals the dimension of death at $\max \{d_i, d_{i+1}\}$, and $t \in \mathbb {N}$ such that $t \in [\min \{d_i,d_{i+1}\}, \max \{d_i, d_{i+1}\})$, but instead, we have $d_i > d_{i+1}$, a symmetric argument shows

$$\begin{aligned} \beta _{k,i,t}^{(1)}&= \beta _{k,i,t}^{(2)} - 1. \end{aligned}$$

Now that we have determined all values of $\beta _{k,s,t}^{(1)}$ and $\beta _{k,s,t}^{(2)}$, we evaluate the sum in Equation (B7). Again, we proceed by cases. In the first case, if *k* does not equal the dimension of death at $\max \{d_i, d_{i+1}\}$, then for all $(s', t')\in \overline{\mathbb {R}}^2$, we have $\mu _k^{(1)}(s', t') = \mu _k^{(2)}(s',t')$. Then, suppose instead that *k* does equal the dimension of death at $\max \{d_i, d_{i+1}\}$. Note that because $f_1$ and $f_2$ are compatible with *f*, we have $f(f^{-1}_1)=f(f^{-1}_2)$. Furthermore, the sets $S = \{s \text { s.t. }f(f_1^{-1}(s)) = f(f_2^{-1}(s)) = s'\}$ and $T = \{t \text { s.t. }f(f_1^{-1}(t)) = f(f_2^{-1}(t)) = t'\}$ are each sets of consecutive integers (with the exception of the possible set $T = \{\infty \}$). We label elements $S = \{s_1, s_2 \ldots , s_r\}$, and write $s_0 = s_1-1$. By a telescoping argument, for any fixed $t \in T$, we find

$$\begin{aligned} \sum _{s \in S}&\left( \beta _{k,s,t-1}^{(1)} - \beta _{k,s,t}^{(1)} - \beta _{k,s-1,t-1}^{(1)} + \beta _{k,s-1,t}^{(1)} \right) \\&= \left( \beta _{k,s_1,t-1}^{(1)} - \beta _{k,s_1,t}^{(1)} - \beta _{k,s_0,t-1}^{(1)} + \beta _{k,s_0,t}^{(1)} \right) \\&+ \left( \beta _{k,s_2,t-1}^{(1)} - \beta _{k,s_2,t}^{(1)} - \beta _{k,s_1,t-1}^{(1)} + \beta _{k,s_1,t}^{(1)} \right) \\&\qquad \vdots \\&+ \left( \beta _{k,s_{r-1},t-1}^{(1)} - \beta _{k,s_{r-1},t}^{(1)} - \beta _{k,s_{r-2},t-1}^{(1)} + \beta _{k,s_{r-2},t}^{(1)} \right) \\&+ \left( \beta _{k,s_r,t-1}^{(1)} - \beta _{k,s_r,t}^{(1)} - \beta _{k,s_{r-1},t-1}^{(1)} + \beta _{k,s_{r-1},t}^{(1)} \right) \\&= - \beta _{k,s_0,t-1}^{(1)} + \beta _{k,s_0,t}^{(1)} + \beta _{k,s_r,t-1}^{(1)} - \beta _{k,s_r,t}^{(1)}, \end{aligned}$$

and similarly for $\beta _{k,s,t}^{(2)}$ when considering $f_2$. We will show that these sums for $f_1$ and $f_2$ agree for any choice of *t*.

Suppose for the fixed *t* chosen above, $t \ge \max \{d_i, d_{i+1}\}$ or $t < \min \{d_i,d_{i+1}\}$ and $t-1 \ge \max \{d_i, d_{i+1}\}$ or $t -1 < \min \{d_i,d_{i+1}\}$. For these values of *t*, we know

$$\begin{aligned} -\beta _{k,s_0,t-1}^{(1)} + \beta _{k,s_0,t}^{(1)} + \beta _{k,s_r,t-1}^{(1)} - \beta _{k,s_r,t}^{(1)} = -\beta _{k,s_0,t-1}^{(2)} + \beta _{k,s_0,t}^{(2)} + \beta _{k,s_r,t-1}^{(2)} - \beta _{k,s_r,t}^{(2)}. \end{aligned}$$

Suppose for the fixed *t* chosen above, both $t, t-1 \in [\min \{d_i, d_{i+1}\}, \max \{d_i, d_{i+1}\})$ and $d_i < d_{i+1}$. Then, we know

$$\begin{aligned}&-\beta _{k,s_0,t-1}^{(1)} + \beta _{k,s_0,t}^{(1)} + \beta _{k,s_r,t-1}^{(1)} - \beta _{k,s_r,t}^{(1)} \\&\qquad = -(\beta _{k,s_0,t-1}^{(2)}+1) + (\beta _{k,s_0,t}^{(2)}+1) + (\beta _{k,s_r,t-1}^{(2)}+1) - (\beta _{k,s_r,t}^{(2)}+1) \\&\qquad = -\beta _{k,s_0,t-1}^{(2)} + \beta _{k,s_0,t}^{(2)} + \beta _{k,s_r,t-1}^{(2)} - \beta _{k,s_r,t}^{(2)}. \end{aligned}$$

For the same *t* and $t-1$, if $d_i > d_{i+1}$, a nearly identical argument holds.

If $t = \min \{d_i, d_{i+1}\}$ and $d_i < d_{i+1}$, then we have

$$\begin{aligned}&-\beta _{k,s_0,t-1}^{(1)} + \beta _{k,s_0,t}^{(1)} + \beta _{k,s_r,t-1}^{(1)} - \beta _{k,s_r,t}^{(1)} \\&\qquad = -\beta _{k,s_0,t-1}^{(2)} + (\beta _{k,s_0,t}^{(2)}+1) + \beta _{k,s_r,t-1}^{(2)} - (\beta _{k,s_r,t}^{(2)}+1) \\&\qquad = -\beta _{k,s_0,t-1}^{(2)} + \beta _{k,s_0,t}^{(2)} + \beta _{k,s_r,t-1}^{(2)} - \beta _{k,s_r,t}^{(2)}. \end{aligned}$$

Again, for the same *t* and $t-1$, if $d_i > d_{i+1}$, a nearly identical argument holds.

Note that because we cannot have $t-1 \in [\min \{d_i, d_{i+1}\}, \max \{d_i, d_{i+1}\})$ without *t* also being in this set, we have evaluated all possible cases of *t* and $t-1$. In each case, the term of the sum corresponding to Equation (B7) agreed between $\mu ^{(1)}(s',t')$ and $\mu ^{(2)}(s',t')$. This, combined with our previous arguments, means we have shown the equality $\mu ^{(1)}(s',t') = \mu ^{(2)}(s',t')$, as desired. $\square$

We have, so far, only considered the case that *i* and $i+1$ correspond to birth events. The remaining three cases (i.e., that they correspond to death events, a birth and death event, or a death and birth event) are similar. All cases taken together give us the following corollary.

### Corollary 47

(Single Transposition) Let $K$ be a simplicial complex, $m \in \mathbb {N}$, and $f :K \rightarrow \mathbb {R}$ a filter function. Let $f_1$ and $f_2$ be two *f*-compatible index functions that differ by exactly one transposition. Then:

$$\left\{ \left( f\left( f_1^{-1}(i)\right) , f\left( f_1^{-1}(j)\right) \right) \right\} _{(i,j) \in \mathcal {D}_{f_t}} = \left\{ \big (f(f_2^{-1}(i)), f(f_2^{-1}(j)) \big ) \right\} _{(i,j) \in \mathcal {D}_{f_1}},$$

with $f(\emptyset ):=\infty$ and where $=$ denotes equality as a multi-set.

Next, we move to transposition sequences of arbitrary finite length, and show that all index filtrations in a transposition sequence produce the same verbose persistence diagram.

### Lemma 48

(Transposition) Let $K$ be a simplicial complex, $m \in \mathbb {N}$, and $f :K \rightarrow \mathbb {R}$ a filter function. Let $\{f_1,f_2,\ldots , f_m\}$ be an *f*-compatible transposition sequence. Then, for all $t \in \{ 1,2, \ldots , m\}$,

$$\left\{ \left( f\left( f_t^{-1}(i)\right) , f\left( f_t^{-1}(j)\right) \right) \right\} _{(i,j) \in \mathcal {D}_{f_t,k}} = \left\{ \big (f(f_1^{-1}(i)), f(f_1^{-1}(j)) \big ) \right\} _{(i,j) \in \mathcal {D}_{f_1,k}},$$

with $f(\emptyset ):=\infty$ and where $=$ denotes equality as a multi-set.

### Proof

For $t \in \{1,2, \ldots , m\}$, we write

B8 $$\begin{aligned} {A}_t := \left\{ \left( f \left( {f}_t^{-1}(i)\right) , f\left( {f}_t^{-1}(j)\right) \right) \right\} _{(i,j) \in \mathcal {D}_{f_t}}. \end{aligned}$$

We prove the general claim by induction on *m*, the length of *f*-compatible transition sequence.

The base case is straightforward: Let $f_1$ be an *f*-compatible index filtration. We immediately have $A_1=A_1$.

For the inductive assumption, let $m \ge 1$, and suppose for any *f*-compatible transposition sequence, $\{f_1,f_2, \ldots , f_m \}$, we have $A_{m'}=A_1$ for all $m' \in \{1,2, \ldots , m \}$.

Now, consider $\{f_1,f_2,\ldots , f_{m+1}\}$, an *f*-compatible transposition sequence of length $m+1$. By the inductive assumption, we know that $A_2 = A_3 = \ldots = A_{m+1}$, so it suffices to show that $A_1 = A_{2}$.

Recall that $f_1$ and $f_{2}$ differ by one transposition, so let $\sigma$ and $\tau$ be the simplices transposed and suppose the transposition occurs at indexes *i* and $i+1$. By Corollary 47, we have $A_1 = A_2$. Thus, we have shown the claim. $\square$

Finally, we show all index filtrations compatible with a common underlying filtration all produce the same VPD, i.e., VPDs are well-defined.

### Lemma 49

(VPD is Well-Defined) The verbose persistence diagram is well-defined.

### Proof

Let $K$ be a simplicial complex, and let $f :K\rightarrow \mathbb {R}$ be a filter function.

Let $f', f'' :K\rightarrow \mathbb {R}$ be two index filters compatible with *f*. To show that the VPD is well-defined, we show that $f'$ and $f''$ produce the same VPD.

Because $f'$ and $f''$ are compatible with *f* and produce a total order on the simplices of $K$, there exists a sequence of filters $\{ f_1,f_2, \ldots , f_m \}$ such that $f_1=f'$, $f_m=f''$, and consecutive filter functions agree except for one *f*-compatible transposition. As in Equation (3) of Definition 1, define the following multisets for $t \in \{1,2,\ldots , m\}$:

B9 $$\begin{aligned} {A}_t := \left\{ \left( f \left( {f}_t^{-1}(i)\right) , f\left( {f}_t^{-1}(j)\right) \right) \right\} _{(i,j) \in \mathcal {D}_{f_t}}. \end{aligned}$$

By Lemma 48, we know that $A_t=A_1$ for all *t*. In particular, $A_m=A_1$. Thus, both $f'$ and $f''$ produce the same verbose persistence diagram, which means that VPDs are well-defined. $\square$

To show that VBFs and VECFs are well-defined, we first observe the following relationship between descriptors, arising from the connection between homology, Betti number, and Euler characteristic.

### Observation 50

Given the verbose persistence diagram $\widetilde{\mathcal {D}}_{f}$ for some simplicial complex $K$ and filter function $f:K\rightarrow \mathbb {R}$, we know the Betti numbers at every step of a corresponding index filtration and can thus construct the verbose Betti function $\widetilde{\beta }_{f}$. Furthermore, we can construct the verbose Euler characteristic function $\widetilde{\chi }_f$ by taking the alternating sum of these Betti numbers.

Then, well-definedness of both the VBF and VECF are straightforward corollaries of Lemma 49; because two distinct index filters compatible with the same filter function produce the same VPD, they produce the same VBF and the same VECF.

### Corollary 51

(VBF and VECF are Well-Defined) Let $K$ be a simplicial complex and let $f :K\rightarrow \mathbb {R}$ be a filter function. Then, the verbose Betti function $\widetilde{\beta }_{f}$ and the verbose Euler characteristic function $\widetilde{\chi }_f$ are well-defined.

## Proof of Simplex Count Lemma

In this appendix, we provide the proof of Lemma 6. We first recall the statement of this well-known result.

### Lemma of 6

(Simplex Count) Let $K$ be a simplicial complex, $k \in \mathbb {N}$, and $c \in \mathbb {R}$. Let $f :K\rightarrow \mathbb {R}$ be a filter function. Then, the *k*-dimensional simplices of $K$ with a function value of *c* are in one-to-one correspondence with the points in the following multiset:

9 $$\begin{aligned} \left\{ (a,b) \in \widetilde{\mathcal {D}}_{f, k} \text { s.t. } a = c \right\} \sqcup \left\{ (a,b) \in \widetilde{\mathcal {D}}_{f, k-1} \text { s.t. } b = c \right\} . \end{aligned}$$

### Proof

Let $f' :K\rightarrow \mathbb {N}$ be an index filter function compatible with *f*, and let $\{F'_i \}_{i \in \mathbb {N}}$ be the corresponding index filtration, where $F'_i := f'^{-1}(-\infty ,i]$. Let $\sigma _1,\sigma _2, \ldots , \sigma _n$ be the ordering of simplices in *K* such that $f'(\sigma _i)=i$.

Consider the sets

$$\begin{aligned} C_B := \left\{ (i,j) \in \mathcal {D}_{f',k} \text { s.t. } f(\sigma _i) = c \right\} \quad C_D := \left\{ (i,j) \in \mathcal {D}_{f',k-1} \text { s.t. } f(\sigma _j)= c \right\} \end{aligned}$$

and let $$C= C_B \cup C_D$$.

We start by defining a bijection $\phi :f^{-1}(c) \rightarrow C$. Let $\sigma _i \in K_k$ such that $f(\sigma _i)=c$. Because adding a single *k*-simplex either increases $\beta _k$ by one or decreases $\beta _{k-1}$ (see, e.g., [12, pp. 120–121]), either $\beta _k(F'_{i})=\beta _k(F'_{i-1})+1$ or $\beta _{k-1}(F'_{i})=\beta _{k-1}(F'_{i-1})-1$, but not both.

1. ($\beta _k$ increases). There exists a unique birth at index *i* in $\mathcal {D}_{f',k}$. Thus, let $j \in \overline{\mathbb {R}}$ be such that $(i,j) \in \mathcal {D}_{f',k}$. Then, $(f'(\sigma _i),f'(\sigma _j))=(i,j) \in C_B$. Define $\phi (\sigma _i)=(i,j)$.
2. ($\beta _{k-1}$ decreases). There is a unique death at index *i* in $\mathcal {D}_{f',k}$. Thus, let $j \in \overline{\mathbb {R}}$ be such that $(j,i) \in \mathcal {D}_{f',k}$. Then, $(f'(\sigma _j),f'(\sigma _i))=(j,i) \in C_D$. Define $\phi (\sigma _i)=((f'(\sigma _j),f'(\sigma _i))=(j,i)$.

In other words, each $\sigma _i$ is mapped to the persistence pair containing $f'(\sigma _i)$. If $\phi (\sigma )=\phi (\tau )=(i,j)$, then, by construction, both $\sigma$ and $\tau$ are the same type of event. WLOG, assume that they are birth events. Then, $f'(\sigma )=f'(\tau )$, which, by injectivity of $f'$ means that $\sigma =\tau$. Thus, we have shown that $\phi$ is an injection.

We show that $\phi$ is a surjection by contradiction. Suppose there exists $(i,j) \in C$ such that there does not exist $\sigma \in f^{-1}(c)$ with $\phi (\sigma )=(i,j)$. Because $(i,j)\in C$, then either $f(\sigma _i)=c$ or $f(\sigma _j)=c$ (or both). WLOG, suppose $f(\sigma _i)=c$. Then, we find $\sigma _i \in f^{-1}(c)$ and, by construction, $\phi (\sigma _i)=(i,j)\in C_B$.

By Equation (3) and because $\widetilde{\mathcal {D}}_{f}$ is well-defined (see Lemma 49), we know that the map $\psi :\mathcal {D}_{f',k} \rightarrow \widetilde{\mathcal {D}}_{f, k}$ defined by $\psi (i,j)=(f(\sigma _i),f(\sigma _j))$ is a bijection. Because $C \subset \mathcal {D}_{f',k}$ and both functions are bijections, the composition $\psi \circ \phi$ is the one-to-one correspondence that we sought. $\square$

Thus, if some step in a filtration includes a single additional simplex, there must be a single additional point in the diagram. This result is noted elsewhere, e.g., [12, Chapter V.4], and we state it as a corollary to Lemma 6.

### Corollary 52

(Add One Simplex) Let $K_i$ and $K_{i+1}$ be two simplicial complexes such that $K_{i+1} \setminus K_i$ is a single *k*-simplex. Let $\beta _k^{(i)}$ and $\beta _k^{(i+1)}$ denote the *k*th Betti numbers of $K_i$ and $K_{i+1}$, respectively. Then, either $\beta _k^{(i+1)} = \beta _k^{(i)} + 1$ or $\beta _{k-1}^{(i+1)} = \beta _{k-1}^{(i)} - 1$. No other Betti numbers change values.

## Omitted Algorithms and Proofs

In this appendix, we provide algorithms and proofs omitted from the main text.

### Proofs from Section 4.5

We begin with the proof of Theorem 25, which we restate below.

#### Theorem of 25

(Computing Even/Odd-Indegree) Let $K$ be a simplicial complex GP-immersed in $\mathbb {R}^d$. Let $\sigma$ be a simplex with $\textrm{verts}(\sigma ) \subseteq K_0$ and $s\in \mathbb {S}^{d-1}$ such that $s$ is $K_0$-perpendicular to $\sigma$. Then, for $k \ge \dim (\sigma )$, $\mathtt{{EvenIndeg}}(\sigma , s,\widetilde{\chi }[K,S])$ returns the even-indegree of $\sigma$ in direction $s$. Similarly, the odd-version of this algorithm, $\mathtt{{OddIndeg}}(\sigma , s,\widetilde{\chi }[K,S])$ returns the odd-indegree of $\sigma$ in direction $s$.

#### Proof

We begin by considering even-indegree. The proof is similar to the proof of Theorem 21. Because *S* is assumed to be simplex-isolating for *K*, if *S* is not simplex-isolating for $\sigma$, then $\sigma$ is not a simplex of *K* and thus must have even-indegree 0, which is the value returned on Line 2.

Suppose then that *S* is simplex-isolating for $\sigma$. We prove the claim inductively on $j= \dim (\sigma )$ and let $h_s: K \rightarrow \mathbb {R}$ denote the filter function for direction *s*. For the base case ($j=0$), consider $\sigma = [v]$, a single zero-simplex. Because $s$ is $K_0$-perpendicular to $\sigma$, the even-indegree of *v* is exactly equal to the number of even-simplices that have height $s\cdot v$, which can immediately be read off of $\widetilde{\chi }_{h_s}$. In Algorithm 3, notice that because the vertex *v* does not have any proper faces, we do not enter the loop that starts on Line 8. Thus, the return value is exactly the number of even simplices with height $s\cdot v$.

For the inductive step, let $j \ge 0$. We assume that Algorithm 3 returns the even-indegree of $\tau$ in direction $s$, for all $\tau \in K_j$. Suppose that $\dim (\sigma ) = j+1$. Now, we compute the even-indegree of $\sigma$ in direction $s$. Let $F_\sigma$ denote the set of even-dimensional simplices with height $h_s(\sigma )$ in direction $s$, whose cardinality is reported in $\widetilde{\chi }_{h_s}$. Let $\sigma ' \in F_{\sigma }$ and let $\tau \prec \sigma$. Suppose that $s_\tau$ is a $(K_0, {\sigma }, {\sigma \setminus \tau }, s)$-perturbation. By Lemma 24, we know $\sigma '$ contributes to the even-indegree of $\tau$ in direction $s_\tau$ if and only if $\tau = \sigma \cap \sigma '$.

Recall that we have a nearly identical version of Corollary 20 for the setting of even/odd-indegree, which gives a simplified equation for the even-indegree of $\sigma$:

D10 $$\begin{aligned} \delta - \sum _{\tau \prec \sigma } \delta _\tau , \end{aligned}$$

where $\delta$ is the number of even-simplices at the height of $\sigma$ and $\delta _\tau$ is the even-indegree of $\tau$ in the corresponding tilted directions. In Algorithm 3, allEven is equal to $\delta$, and the values $\delta _{\tau }$ are computed in Line 12. Thus, the return value of Algorithm 3 matches the value of Equation (D10).

The proof for odd-indegree is nearly identical. $\square$

Next, we restate and prove Theorem 26.

#### Theorem of 26

(Sufficient Conditions for Faithful Discretization of the VECT) Let $K$ be a simplicial complex GP-immersed in $\mathbb {R}^d$ such that $\dim (K) < d$ and let $S \subset \mathbb {S}^{d-1}$ such that $(K,S)$ is vertex- and simplex-isolating. Then, Algorithm 4 reconstructs *K*, so $\widetilde{\chi }[K,S]$ is a faithful discretization of $\widetilde{\chi }[K,\mathbb {S}^{d-1}]$.

#### Proof

The initial steps of the proof follow the reasoning given in Theorem 22; we reconstruct $K_0$, and when we consider some *V* with $|V |-1 = k$, we have already reconstructed $K_{k-1}$. First, suppose *k* is even. Because $\widetilde{\mathcal {D}}[K,S]$ is simplex-isolating, if *V* defines a simplex of *K*, the set *S* contains a direction *s* that is $K_0$-perpendicular to $\textrm{aff}(V)$. Thus, if there is no such direction, *V* does not define a simplex of *K*.

If there is such a direction, by Theorem 25, $\mathtt{{EvenIndeg}}(\sigma , s, \widetilde{\chi }[K,S], T = \{ \})$ (Algorithm 3) returns the number of even-simplices at the height of $\textrm{aff}(V)$ that contain $\sigma$ as a face; if this value is 0, we know *V* is not a simplex of *K*. If this value is 1 or greater, we know *V* does define a simplex of *K*, and we add *V* to $K_k$. A nearly identical argument holds for the case that *k* is odd. Because we iterate over all subsets of $K_0$, the algorithm eventually finds all simplices.

Finally, because we have shown $\mathtt{{VECFReconstructComplex}}(\widetilde{\chi }[K,S])$ reconstructs *K*, we know $\widetilde{\chi }[K,S]$ is a faithful discretization of $\widetilde{\chi }[K,\mathbb {S}^{d-1}]$. $\square$

### Algorithms and Proofs from Section 5.1

In this appendix, we provide algorithms and proofs that were omitted from Section 5.1.

***Computing a Perpendicular Direction***

#### Lemma 53

(Computing a Perpendicular Direction) Algorithm 8 is correct and runs in $\Theta (d^3)$ time.

#### Proof

Denote the points of *P* by $p_0, p_1, \ldots , p_{k}$. In Line 1, we define *A* as the $d \times k$ matrix consisting of $p_j - p_0$ in column *j*. By Theorem 7.1 in [36], *A* has a reduced QR-factorization in which the columns of *Q* are an orthonormal basis for the space spanned by *A*, or $\textrm{aff}(P)$. We find *Q* using *k* iterations of Gram–Schmidt on Line 2.

Then, in the loop at Lines 5–8, we iteratively test the result of performing Gram–Schmidt on the columns of *Q* and a basis vector. The result is zero if the basis vector is contained in $\textrm{aff}(P)$. As soon as the result is nonzero, (which has to happen because $\textrm{aff}(P)$ has positive codimension, so can’t be spanned by all $e_i$s), we have found a vector orthogonal to $\textrm{aff}(P)$, and the value returned on Line 9 is as desired.

Building the matrix *A* and normalizing $s$ takes constant time. Finding the reduced QR-factorization takes *k* iterations of Gram–Schmidt, (see [36, Theorem 8.1]) and finding the final vector that is orthogonal to the space spanned by *P* requires at most *d* iterations of Gram–Schmidt. Because each iteration of Gram–Schmidt takes *O*(*dk*) time, we see that the total runtime is $\Theta (d^3)$. $\square$

***Plane Filling***

#### Lemma 54

(Plane Filling) Algorithm 9 is correct and runs in $\Theta (d^3)$ time.

#### Proof

In Lines 2 and 3 of Algorithm 9, we find an orthonormal basis for the space spanned by $\{p_1-p_0, p_2-p_0, \ldots , p_k-p_0,s\}$ in $\Theta (kd)$ time, following the same process we did in Lines 1 and 2 of Algorithm 8; see proof of Lemma 53.

Then, by using Gram–Schmidt in Lines 5–13, we find $d-(k+1)$ vectors spanning the space orthogonal to $\textrm{aff}(A)$, taking a total of $\Theta (d^3)$ time. While doing so, we also iteratively add points to $P'$ in Line 9. Computing the full QR-decomposition and performing Gram–Schmidt dominates the algorithm; hence, the running time for Algorithm 9 is $\Theta (d^3)$.

Finally, for correctness, we prove that the output of Algorithm 9 has the desired properties. By construction, we have $|P'|= d - |P |$, i.e., $\dim (\textrm{aff}(P' \cup P)) = d-1$. To show each point in $P'$ is at the same height as *P*, it suffices to show that $s\cdot p_0 = s\cdot p'$ for all $p' \in P'$. Because each $s_\ell$ considered in Line 9 is in the basis for the left null space of *A* and *s* is a column of *A*, all such vectors $s_\ell$ are orthogonal to $s$. Then, indeed, for all $p' \in P'$, we have $s\cdot p' = s\cdot (s_\ell + p_0) = s\cdot p_0$. $\square$

## Example of Building a Faithful Set

Here, we walk through a small example of building a faithful discretization of the VPHT, VBT, or VECT, following Construction 34. Suppose we are given the simplicial complex *K* in $\mathbb {R}^3$, shown in Fig. 7. Specifically, *K* is: five vertices, $v_0 = (1,0,0)$, $v_1 = (0,1,0)$, $v_2 = (0,0,1)$, $v_3=(0,2,0)$, and $v_4=(1,0,2)$; four edges, $[v_0,v_1]$, $[v_1,v_2]$, $[v_0,v_2]$, and $[v_0,v_3]$; and a two-simplex $[v_0,v_1,v_2]$. There are three maximal simplices, $v_4$, $\sigma _1=[v_0,v_3]$, and $\sigma _2=[v_0,v_1,v_2]$.

### Tilt Examples

Because many algorithms used in Construction 34 make calls to Algorithm 5 ($\mathtt{{Tilt}}$), we walk through two explicit examples in this section, using inputs that are relevant for later subsections.

We first describe the details of the call $\mathtt{{Tilt}}(s,s',P)$ where $s=(1,0,0)$, $s'=(0,1,0)$ and $P = \{0,1,2\}^3 \subset \mathbb {R}^3$. We begin by finding the heights of points in *P* with respect to the direction *s* that are closest but distinct (Line 4 of Algorithm 5). There are only two distinct heights of points of *P* with respect to the direction *s*, namely 0 and 1, so we have $h_1 = 0$ and $h_2 = 1$. Next, on Line 5, we find heights of points of *P* in the direction $s'$ that are extremal, which are $h_1' = 0$ and $h_2' = 2$. Then, on Line 6, we compute the solution to $(1-t) h_1 + t h_2' = (1-t)h_2 + t h_1'$, which is $t_* = 1/3$. Finally, we return the tilted direction

$$\begin{aligned} s_t = \left( 1-\frac{1}{2}t_*\right) e_1+ \frac{1}{2}t_* e_2 = \left( \frac{5}{6}, \frac{1}{6}, 0\right) \approx (0.8333, 0.1667, 0). \end{aligned}$$

Next, we detail a more involved instance of $\mathtt{{Tilt}}$, using $s = (2\sqrt{5}/5, \sqrt{5}/5, 0)$, $s' = (0.8, 0.4, 0.4472)$, and $P = K_0$. First, on Line 4, we identify two heights of vertices with respect to $s$ that are closest. The direction $s$ orders vertices in an evenly spaced way ($v_0, v_3,$ and $v_4$ have height $2 \sqrt{5}/5$, $v_1$ has height $\sqrt{5}/5$, and $v_2$ has height 0). Going through the sorted list, we first encounter $h_1 = 0$ and $h_2 = \sqrt{5}/5$. On Line 5, we find the minimum and maximum heights with respect to $s'$. These are $h_1' = 0.4$ (the height of $v_1$ with respect to $s'$) and $h_2' \approx 1.6944$ (the height of $v_4$ with respect to $s'$). Finally, on Line 6 of Algorithm 5, we find the solution to $(1-t) h_1 + t h_2' = (1-t)h_2 + t h_1'$, which is $t_* \approx 0.2568$. This gives us the final output of $\mathtt{{Tilt}}(s,s',K_0)$,

$$\begin{aligned} s_t = \left( 1-\frac{1}{2}t_*\right) s+ \frac{1}{2}t_* s' \approx (0.8823, 0.4412, 0.0574). \end{aligned}$$

### Vertex Isolating Directions

First, we find the directions of Step 1 of Construction 34. Specifically, we find directions that are vertex-isolating with respect to *K*. By Lemma 32, the standard basis directions, $e_1, e_2, e_3$, as well as a fourth direction as computed in $\mathtt{{PointIso}}(K_0)$ (Algorithm 7) are vertex-isolating with respect to *K*. Thus, we walk through the computation of Algorithm 7. First, on Line 1, we begin by defining *A*, the filtration grid of $K_0$ with respect to $\{e_1, e_2, \ldots , e_d\}$. In our example, *A* is a set of 27 gridpoints, $\{0,1,2\}^3 \subset \mathbb {R}^3$. In Line 2, we initialize $s = (1,0,0)$, and enter the loop on Lines 3–5. The first iteration calls $\mathtt{{Tilt}}((1,0,0),(0,1,0),A)$ (Algorithm 5), tilting $e_1$ towards $e_2$. As detailed in Section E.1, this gives us $s^2 = (1-\frac{1}{2}t_*)e_1+ \frac{1}{2}t_* e_2 \approx (0.8333, 0.1667, 0)$. We are then ready for the next and final iteration of the loop in Algorithm 7, finding the fourth direction in our set of vertex-isolating directions,

E11 $$\begin{aligned} s = \mathtt{{Tilt}}(s^2,(0,0,1),A) \approx (0.8013, 0.1603, 0.0385). \end{aligned}$$

### Perpendicular Directions

Next, we compute directions as described in Step 2a of Construction 34 (equivalently, directions satisfying Statement (1) of Definition 12). Then, in the language of Definition 12, for $\sigma _i$ ($i = 1,2$) we need a direction $s_{\sigma _i}$ that is $K_0$-perpendicular to $\sigma _i$. We begin with the two-simplex, $\sigma _2$, the more straightforward computation. First, we compute $\mathtt{{Perp}}(\sigma _2)$ (Algorithm 8). In Line 1 of Algorithm 8, we find a matrix *A* with $v_j - v_0$ in the *j*th column, which, for $\sigma _2$ is

E12 $$\begin{aligned} A = \begin{bmatrix} -1 &  -1 \\ 1 &  0 \\ 0 &  1 \end{bmatrix}. \end{aligned}$$

Next, in Line 2, we use a QR-decomposition to find the basis vectors for the space spanned by *A*, which are, $b_1 = (-\sqrt{2}/2,\sqrt{2}/2,0)$ and $b_2 = (-\sqrt{6}/6, -\sqrt{6}/6, \sqrt{6}/3)$. Then, in Lines 5–8, we perform Gram-Schmidt on $b_1$ and $b_2$ with each basis vector until the result is non-zero. The loop terminates after one iteration and we find

E13 $$\begin{aligned} s_{\sigma _2} = \mathtt{{Perp}}(\sigma _2) = (\sqrt{3}/3, \sqrt{3}/3, \sqrt{3}/3) \approx (0.5774, 0.5774, 0.5774). \end{aligned}$$

The direction $s_{\sigma _2}$ is already $K_0$-perpendicular to $\sigma _2$; we confirm this by completing the procedure suggested in Step 2a of Construction 34. Let *V* be the set of vertices with the same height as $\sigma _2$ in direction $s_{\sigma _2}$, meaning $V = \textrm{verts}(\sigma )$. Then, we set $W = V \setminus \sigma = \emptyset$, so $\mathtt{{TiltToPop}}(K_0,V,W,s_{\sigma _2}) = \mathtt{{TiltToPop}}(K_0,\sigma _2,\emptyset ,s_{\sigma _2}) = s_{\sigma _2}$ (this is highlighting the fact that, because the output of $\mathtt{{Perp}}(\sigma _2)$ is already $K_0$-perpendicular to $\sigma _2$, we do not need to adapt it in any way).

Next, we shift focus to $\sigma _1$. To construct the direction described by Step 2a of Construction 34 for the edge $\sigma _1$, we again begin by finding $\mathtt{{Perp}}(\sigma _1)$. This begins with the column matrix containing $v_3 - v_0$,

E14 $$\begin{aligned} A_1 = \begin{bmatrix} -1 \\ 2 \\ 0 \end{bmatrix}. \end{aligned}$$

Then, we use QR-decomposition to find the basis for the space spanned by $A_1$, which consists of the single vector $b = (-\sqrt{5}/5, 2\sqrt{5}/5, 0)$. Performing Gram-Schmidt on *b* and $e_1$ yields $s= \mathtt{{Perp}}(\sigma _1) = (2\sqrt{5}/5, \sqrt{5}/5, 0)$. However, note that although $s$ is perpendicular to $\sigma _1$, it is not $K_0$-perpendicular, because the vertex $v_4$ is at the same height as $\sigma _1$ with respect to $s$. Thus, we correct $s$ by following the procedure suggested in Construction 34, Step 2a. First, let $V_1$ be the set of all vertices with the same height as $\sigma _1$ in direction $s$, i.e., $V_1= \{v_0, v_3, v_4\}$. We also set $W_1 = V_1 \setminus \sigma _1 = \{v_4\}$. Then we use $\mathtt{{TiltToPop}}(K_0,V_1,W_1,s)$ (Algorithm 6) to find a direction $K_0$-perpendicular to $\sigma _1$. In Line 1, we find $V' = \mathtt{{PlaneFill}}(V_1,s)$ but because $\textrm{aff}(V_1)$ has codimension one with $\mathbb {R}^3$, we simply have $V' = \emptyset$. Next, in Line 2, we compute

$$\begin{aligned} s'&= \mathtt{{Perp}}({V' \cup (V_1 \setminus W_1) \cup \{W_1 - s\}}) \\&= \mathtt{{Perp}}({v_0, v_3, v_4 - s}) \\&= \mathtt{{Perp}}({v_0, v_3, (1-2\sqrt{5}/5, -\sqrt{5}/5, 2)}) \\&\approx (0.8, 0.4, 0.4467). \end{aligned}$$

Although for this example, $s'$ happens to be already $K_0$ perpendicular to $\sigma _1$, this is not generally the case. Thus, we continue on Line 6 and compute

$$\begin{aligned} s_{\sigma _1} = \mathtt{{Tilt}}(s,s',K_0 \cup V') = \mathtt{{Tilt}}(s,s',K_0) \approx (0.8929, 0.4465, 0.0581) \end{aligned}$$

(Algorithm 5, see Section E.1 for details). This $s_{\sigma _1}$ is then the output of $\mathtt{{TiltToPop}}( ,K_0,V_1){W_1}{s}$, and a direction guaranteed to be $K_0$-perpendicular to $\sigma _1$.

### Simplex-Isolating Directions

Next, we find the directions of Step 2b in Construction 34 (directions satisfying Statement (2) of Definition 12). That is, for every nonempty proper face of $\sigma _1$ and $\sigma _2$, we need a direction that pops the face above the remaining vertices of the maximal simplex. We walk through the procedure for a single face of $\sigma _2$; the remaining computations are similar and are summarized in Table 1.

Consider the vertex $[v_0] \prec \sigma _2$. By Theorem 35, we can use $\mathtt{{TiltToPop}}(K_0,\sigma _2, ){v_0}{s_{\sigma _2}}$ (Algorithm 6) to find a direction that pops $v_0$ above $\sigma _2 \setminus v_0 = [v_1,v_2]$. First, in Line 1, we find $V' = \mathtt{{PlaneFill}}(\sigma _2,s_{\sigma _2})$ using Algorithm 9. Because $\sigma _2$ has codimension one with $\mathbb {R}^3$, this is simply $V' = \emptyset$. Next, on Line 2 of Algorithm 6 we compute

$$\begin{aligned} s'&=\mathtt{{Perp}}({V' \cup (\sigma _2 \setminus v_0) \cup \{v_2 - s_{\sigma _2}\}}) \\&= \mathtt{{Perp}}({\{v_1,v_2\} \cup \{v_0 - s_{\sigma _2}\}}) \\&\approx (0.9636, 0.1890, 0.1890). \end{aligned}$$

Because $v_0$ is above $[v_1,v_2]$ with respect to $s'$, we do not enter the if loop on Line 3. Finally, we use Algorithm 5 and compute

$$\begin{aligned} s= \mathtt{{Tilt}}(s_{\sigma _2},s',K_0) \approx (0.6598, 0.4944, 0.4944), \end{aligned}$$

which is therefore also the output to $\mathtt{{TiltToPop}}(K_0,\sigma _2,v_0,s_{\sigma _2})$.

***Summary of Computations***

Below is the complete list of computed directions, including those that were discussed in detail previously in this section.

**Table 1:** Directions that are vertex- and simplex-isolating for the simplicial complex of Fig. 7, computed as described in Construction 34

Property	Description	Computed Directions
		(rounded to four decimal places)
Definition 11	vertex-isolating	(1,0,0)
		(0,1,0)
		(0,0,1)
		(0.8013, 0.1603, 0.0385)
Statement (1)	$K_0$-perpendicular to $\sigma _2$	$s_{\sigma _2} = (0.5774, 0.5774, 0.5774)$
of Definition 12	$K_0$-perpendicular to $\sigma _1$	$s_{\sigma _1} = (0.8929, 0.4465, 0.0581)$
Statement (2)	$(K_0, \sigma _2, [v_0], s_{\sigma _2})$-perturbation	(0.6598, 0.4944, 0.4944)
of Definition 12	$(K_0, \sigma _2, [v_1], s_{\sigma _2})$-perturbation	(0.5357, 0.6187, 0.5357)
	$(K_0, \sigma _2, [v_2], s_{\sigma _2})$-perturbation	(0.5357, 0.5357, 0.6187)
	$(K_0, \sigma _2, [v_0,v_1], s_{\sigma _2})$-perturbation	(0.5953, 0.5953, 0.4866)
	$(K_0, \sigma _2, [v_0,v_2], s_{\sigma _2})$-perturbation	(0.5959, 0.4835, 0.5959)
	$(K_0, \sigma _2, [v_1,v_2], s_{\sigma _2})$-perturbation	(0.5235, 0.5880, 0.5880)
	$(K_0, \sigma _1, [v_0], s_{\sigma _1})$-perturbation	(0.8879, 0.4351, 0.0753)
	$(K_0, \sigma _1, [v_3], s_{\sigma _1})$-perturbation	(0.8834, 0.4504, 0.0579)

## Footnotes

1 By *general position* we mean that, for all *k* with $1 \le k \le d+1$, every subset of *V* of size *k* is affinely independent. We denote the affine space spanned by *V* by $\textrm{aff}(V)$. Note that this is a stronger condition than the usual notions of immersions; as long as the vertices are in general position, self-intersections of a GP-immersed complex need not be transverse.

2 In fact, if the vertex locations are known, then we are only using dimension and the partial order of the vertices induced by their heights in the given direction.

3 The stability of the entire PHT follows from the stability of (directional) persistence diagrams under continuous changes to a shape or changing direction. See, e.g., [1, Theorem 4.11].
